# Environmental Factors Support the Formation of Specific Bacterial Assemblages on Microplastics

**DOI:** 10.3389/fmicb.2017.02709

**Published:** 2018-01-19

**Authors:** Sonja Oberbeckmann, Bernd Kreikemeyer, Matthias Labrenz

**Affiliations:** ^1^Biological Oceanography, Leibniz Institute for Baltic Sea Research Warnemuende (IOW), Rostock, Germany; ^2^Institute of Medical Microbiology, Virology and Hygiene, University Medical Center Rostock, Rostock, Germany

**Keywords:** microbial biofilm, bacteria, vector function, microplastics, marine microbiology, environmental factors

## Abstract

While the global distribution of microplastics (MP) in the marine environment is currently being critically evaluated, the potential role of MP as a vector for distinct microbial assemblages or even pathogenic bacteria is hardly understood. To gain a deeper understanding, we investigated how different *in situ* conditions contribute to the composition and specificity of MP-associated bacterial communities in relation to communities on natural particles. Polystyrene (PS), polyethylene (PE), and wooden pellets were incubated for 2 weeks along an environmental gradient, ranging from marine (coastal Baltic Sea) to freshwater (waste water treatment plant, WWTP) conditions. The associated assemblages as well as the water communities were investigated applying high-throughput 16S rRNA gene sequencing. Our setup allowed for the first time to determine MP-dependent and -independent assemblage factors as subject to different environmental conditions in one system. Most importantly, plastic-specific assemblages were found to develop solely under certain conditions, such as lower nutrient concentration and higher salinity, while the bacterial genus *Erythrobacter*, known for the ability to utilize polycyclic aromatic hydrocarbons (PAH), was found specifically on MP across a broader section of the gradient. We discovered no enrichment of potential pathogens on PE or PS; however, the abundant colonization of MP in a WWTP by certain bacteria commonly associated with antibiotic resistance suggests MP as a possible hotspot for horizontal gene transfer. Taken together, our study clarifies that the surrounding environment prevailingly shapes the biofilm communities, but that MP-specific assemblage factors exist. These findings point to the ecological significance of specific MP-promoted bacterial populations in aquatic environments and particularly in plastic accumulation zones.

## Introduction

Microplastics (MP) can be found worldwide in aquatic ecosystems, reaching from populated to pristine, from marine to freshwater regions. Specific MP accumulation zones emerge due to oceanographic currents, such as in the subtropical gyres (Cózar et al., 2014) or, due to anthropogenic point sources, in industrial agglomeration areas (Mani et al., 2015). The ecological implications of this pollution in the different environmental systems can so far not be estimated.

The interaction of MP and microbial communities in marine and freshwater is especially underrepresented in the scientific literature. It is, however, well-established that plastic biofilms contain several “usual suspects,” which are abundant in biofilms on many surfaces in aquatic ecosystems. In the marine environment, these include typical initial colonizers of the family *Rhodobacteraceae* (Dang et al., 2008; Zettler et al., 2013) and the algae group *Bacillariophyta* (Carson et al., 2013; Oberbeckmann et al., 2014; Reisser et al., 2014). Members of the *Campylobacteraceae* were found to be abundant on MP sampled in an urban river in Chicago (USA) (McCormick et al., 2014) as well as on MP incubated in coastal sediment from the Humber Estuary (UK) (Harrison et al., 2014). Following one assumption by Maso et al. (2003), that a harmful algae bloom species was dispersed in the Mediterranean while hitchhiking on macroplastic debris, the question emerged whether also MP can serve as a vector for harmful microorganisms. In 2013, members of the potentially pathogenic genus *Vibrio* were detected to be abundant on a polypropylene (PP) particle from the North Atlantic (Zettler et al., 2013). Since then, MP from WWTPs and MP passing the guts of aquatic organisms have been hypothesized to play a particular role as vector for pathogenic microorganisms (McCormick et al., 2014; Oberbeckmann et al., 2015). Studies on *Arenicola marina* and *Mytilus edulis*, however, did not reveal a distinct enrichment of potential pathogens on PS (Kesy et al., 2016, 2017).

Pioneering studies in the field of MP biofilms reveal that biogeography plays an important role for the composition of MP colonizing communities (Oberbeckmann et al., 2014; Amaral-Zettler et al., 2015). Not much, however, is known about the plastic-colonizing processes on a local to regional scale, and a detailed understanding of the factors which are shaping the composition, activity, and potential specificity of MP-associated assemblages is lacking. No investigation of MP-colonization comprised marine as well as freshwater environments, and only very limited previous research enabled a direct comparisons of MP-attached assemblages with those colonizing natural particles, e.g., marine snow.

Our study addressed these knowledge gaps to be able to reliably evaluate the potential of MP to be a vector of distinct microbial assemblages or even pathogens and to better understand the characteristics and dynamics of microbial MP colonizing communities. Our hypothesis was that environmental factors play a major role for the colonization of natural and synthetic surfaces alike, but that a specific plastic-associated microbiome would develop, different from the microbiomes associated with natural particles. We designed an in *situ* incubation experiment, where PE, PS, and wooden pellets were exposed for 2 weeks at 7 different stations in the coastal Baltic Sea, in the estuary of the river Warnow (Rostock, Germany), and in the effluent basins of a WWTP. Corresponding water communities (free-living and attached to naturally occurring particulate material) were sampled as well. The locations covered different environmental conditions along a salinity and nutrient gradient and included different anthropogenic impacts.

## Experimental procedures

Please see Additional file 1 for more detailed information.

### Experimental design and setup

**Figure 1:**
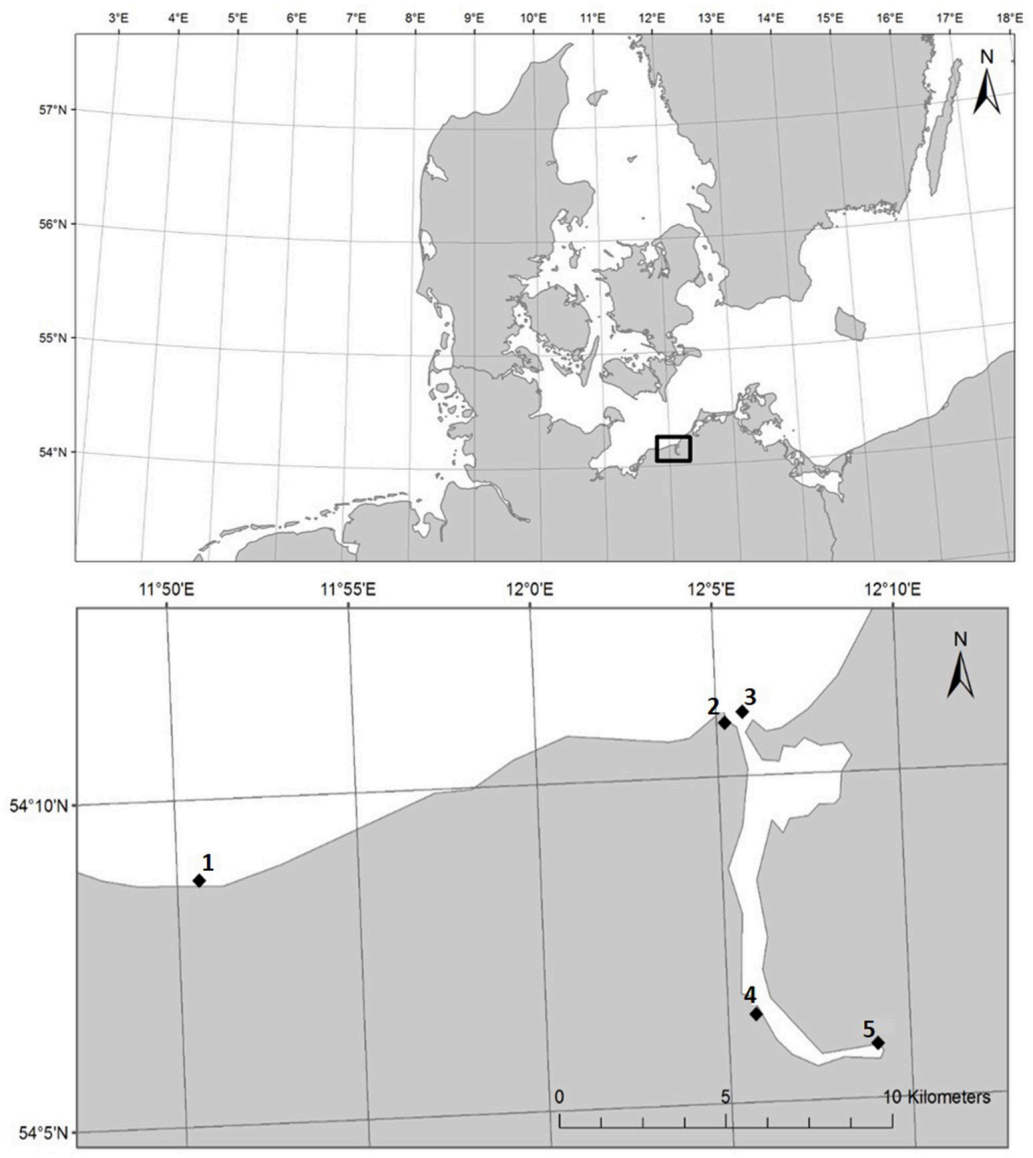
Map section of Northern Europe, with box highlighting sampling area **(Top)**, and individual stations 1-5 **(Bottom)** near Rostock, Germany, from the coastal Baltic Sea to the river Warnow.

HDPE (ExxonMobil^TM^ HDPE HTA 108, density 0.961 g/cm^3^) and PS (BASF Polystyrole 143E, density 1.04 g/cm^3^) pellets (both ø 3 mm) were incubated for 14 days (19./20.08.−03./04.09.2014) at five stations in the estuary of the river Warnow and in the Baltic Sea, Germany (Figure 1). Station 1 was in Heiligendamm (54.146 N 11.843 E), station 2 in the Alter Strom in Warnemünde (54.181 N, 12.087 E), station 3 in the Marine Science Center in Hohe Düne (54.183 N, 12.095 E), station 4 in the Unterwarnow close to a WWTP discharge (54.106 N, 12.096 E), and station 5 in the Unterwarnow close to the city center of Rostock (54.097 N, 12.151 E). As non-plastic control, wooden pellets were incubated. Nine incubators were built in-house and installed in the water column at each station (*n* = 3 per material). Each incubator was covered with a 500 μm pore-sized gauze to keep the pellets inside while assuring sufficient flow-though. Considering the different densities of the materials, 54 g of PS, 50 g of HDPE, or 35 g of wood were added to the incubators. Recovered plastic and wood pellets were rinsed thrice with sterile station water, shock frozen with liquid nitrogen, and stored at −80°C until further processing. To determine whether plastic and wood biofilm communities are unique from surrounding microbial communities, background water samples were collected in triplicate at the end of the 2-week exposure experiment from every station. The >3 μm (1 l, “particle-attached”) and the 3–0.2 μm (300–500 ml, “free-living”) fractions were collected by serial filtration. Filters were shock frozen and stored at −80°C until further analyses.

The experiment was repeated as described above at two stations in a WWTP (28.4.−12.05.2015, name and location remain anonymous as requested by operator). One incubation (station 6) took place in a basin following three treatment steps (a mechanical pre-treatment, an activated sludge, a biological treatment), the other incubation (station 7) following an additional biological filtration, just before the WWTP outflow. Most German WWTPs have a similar setup, releasing the treated water after these 3 or 4 stages.

During both incubation experiments, environmental metadata were collected in triplicates at the beginning and end of the incubation (Table **2**). Temperature and salinity were measured with a portable meter (HQ40d, Hach), while ${\text{PO}}_{4}^{3-}$, ${\text{NO}}_{3}^{-}$, ${\text{NO}}_{2}^{-}$, ${\text{NH}}_{4}^{+}$, and SiO_2_ were determined using a colorimetric method (Grasshoff et al., 1999).

### DNA isolation

DNA was extracted from incubated plastic pellets (20 pellets per sample), wood pellets (1/2 teaspoon), and filters. The procedure involved a treatment with SLS buffer [20% w/v SLS], bead beating and extraction with 1 vol. phenol/chloroform, followed by precipitation using 70% ethanol. Pre-incubated control pellets were subjected to the same procedure.

### Amplification and library generation

Dual-indexing was used to generate a barcoded MiSeq library of tag sequences for each sample of extracted DNA (Kozich et al., 2013). Primers targeting the 16S V4 region were used: 515F (5′ GTGCCAGCMGCCGCGGTAA 3′; (Giovannoni et al., 1988; Caporaso et al., 2011) and 806R (5′ GGACTACHVGGGTWTCTAAT 3′; (Caporaso et al., 2011). Due to low DNA concentrations, a nested PCR approach [with primer pair 27F: 5′ AGAGTTTGATCCTGGCTCAG 3′ (Lane, 1991) and 1492R: 5′ GGTTACCTTGTTACGACTT 3′ Lane, 1991] was applied for samples from station 1–5, while direct amplification could be applied for the samples from station 6 and 7. Sterile water control samples revealed consistently negative results in the nested as well as in the direct amplification approach. Likewise, pre-incubated wood and plastic pellet samples did not lead to a successful amplification using neither the nested nor the direct PCR approach.

### Sequence data processing

Quality filtering of reads (adapter, barcode, primer clipping, permitted length = 250–275 bp, max. number of ambiguous bases per sequence = 0, max. number of homopolymers per sequence = 8), taxonomy assignment (Wang classification, reference database SSURef_123_SILVA, required bootstrap value ≥ 85%) and picking of operational taxonomic units, OTUs (label = 0.03), were carried out using Mothur (Schloss et al., 2009). Chloroplasts, mitochondria, eukaryotes and unknown sequences were removed. OTUs with a total abundance of ≤ 3 were excluded from downstream analyses. All raw sequence files, including sequencing controls, are available from the NCBI Short Read Archive (SRA) database (BioProject PRJNA338729; BioSamples SAMN05567827-SAMN05567879, SAMN06883266, SAMN06883268).

### Alpha and beta diversity and statistical analysis

Chao1 (species richness; Chao, 1984), Shannon H (diversity; Shannon, 1948) and Pielou (evenness; Pielou, 1969) indices were calculated based on a subset of 500 reads per samples (100 iterations). Alpha diversity analyses were carried out with RStudio (v 3.0.2 RStudio Team, 2015) and the vegan package (Oksanen et al., 2015). Using SigmaPlot (v 10, Systat Software GmbH), results of alpha diversity analyses were visualized, and it was tested for significant differences between richness, diversity, and evenness between the three datasets (*t*-test).

For beta diversity analyses, samples with less than 10,000 reads were excluded. Relative abundances of OTUs were calculated and square root transformed. The dataset “Baltic” consisted of samples from stations 2 and 3, the “Warnow” dataset of samples from stations 4 and 5, and the “WWTP” dataset of samples from stations 6 and 7. This segmentation of the data was based on an hierarchical clustering of the environmental factors (**Figure 3**). Station 1 was omitted for the dataset segmentation to assure a similar number of samples per dataset. The “plastic” dataset contained all data on PE and PS associated communities. In our study, the factor “environment” refers to the different sample locations (1–7), the factor “source” refers to the different sample types (PE, PS, wood, free-living, and particle attached water fraction).

Permutational multivariate analysis of variance (PERMANOVA) (Anderson, 2001) was applied to test for significant differences between environments and sources based on Bray-Curtis similarity matrices. *P*-values were obtained using type III sums of squares and 999 permutations. When analyzing solely subsets of the whole dataset, Monte Carlo permutations were calculated due to the lower number of possible permutations. To test whether significant (*p* < 0.05) PERMANOVA results were based on location or dispersion effects, homogeneity of dispersion (PERMDISP) was applied based on calculated distances to centroids (Anderson and Walsh, 2013). To disclose false discovery rates, Benjamini-Hochberg corrections (Benjamini and Hochberg, 1995) for multiple testing was applied using RStudio v 3.3.1 (RStudio Team, 2015). To visualize the patterns of samples regarding environment and source, PCO was performed (Gower, 1966).

The environmental data were normalized and used to construct a Euclidian distance matrix that was subjected to hierarchical clustering (group average), omitting SiO_2_ due to a missing value. CLUSTER, PERMANOVA, PERMDISP, and PCO were calculated with the software PRIMER 6 including the PERMANOVA+ add-on package (PRIMER-E, UK; Clarke and Gorley, 2006; Anderson et al., 2008).

To identify taxa that discriminated (i) the plastic-attached from the other communities, and (ii) the PS from the PE associated communities, the linear discriminant analysis effect size method (LEfSe; Segata et al., 2011) was used. This was performed with the LEfSe online tool in the Galaxy framework, using all default settings for data formatting and LDA effect size. Instead of using a square root transformation, non-transformed relative abundances were used and a LEfSe-internal per-sample normalization was performed. Factor “source” was set as class, and the strategy “all-against-all” was applied. Solely OTUs with a mean (across the whole dataset) relative abundance of ≥0.1% were considered.

A phylogenetic tree was built with the 175 OTUs displaying a mean relative abundance of ≥0.1%. The tree was constructed using mothur, and visualized using the online tool iTOL version 3.2.4 (Letunic and Bork, 2016).

### Quantitative PCR (qPCR)

Standard DNA for *Vibrio*-targeting qPCR originated from an in house culture of *V. aestuarianus* and was extracted using the QIAGEN® QIAamp® DNA Mini Kit. A 114 bp long DNA fragment was amplified using the primer pair 567F (GGCGTAAAGCGCATGCAGGT) and 680R (GAAATTCTACCCCCCTCTACAG) (Thompson et al., 2004), applying an annealing temperature of 60.2°C. Cloning of the purified PCR products into the vector took place with the StrataClone Blunt PCR Cloning Kit (Agilent Technologies, USA) and extraction of the vector from colonies with the QIAGEN® Plasmid Mini Kit (QIAGEN, DE). The samples were analyzed in three qPCR runs, each run including three standard samples. The mean values of the run conditions are listed in the following: slope = 3.155 ± 0.062, PCR efficiency = 107.53 ± 2.97, γ intercept = 45.609 ± 1.440, *R*^2^ = 0.998±0.002, Ct cut-off = 30.7.

## Results

### The environment determines the substrate specificity of microbial colonization

**Figure 2:**
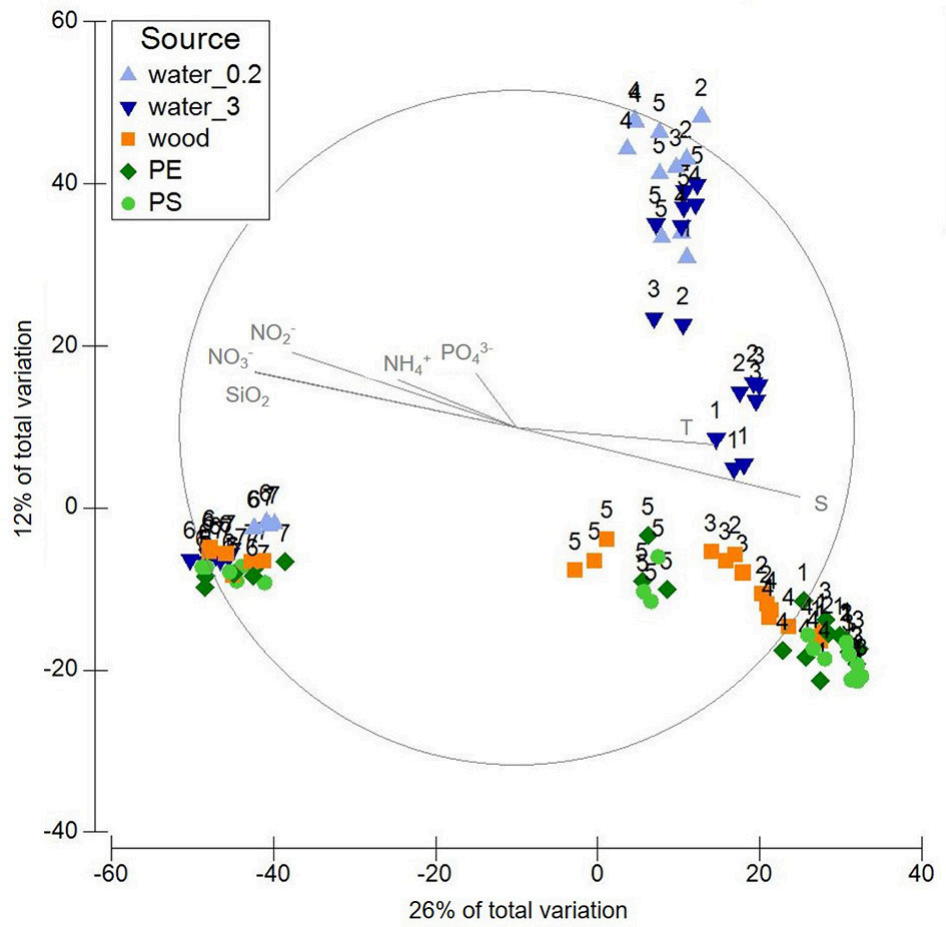
PCO displaying variation in microbial community composition in the different sample sources water (free-living fraction=water_0.2; particle-attached fraction=water_3), wood, PE, PS. Labels refer to sampling stations 1-7. Plot is based on a Bray-Curtis similarity matrix of square root transformed relative abundances of OTUs (16S rRNA gene data).

To verify the overall statistical relevance of the factors “environment” and “source,” PERMANOVA was carried out, and the community patterns were visualized using principal coordinates analysis (PCO, Figure 2).

The factor “environment” differentiated the communities significantly (global *p* = 0.001; 20 of 21 pairwise *p* < 0.05, Additional file 2). The factor “source” (PE, PS, wood, water free-living, and particle-attached) also differentiated the communities significantly (global *p* = 0.001; 9 of 10 pairwise *p* < 0.05, Table 1). Solely the communities on PE and PS did not differ significantly. Benjamini-Hochberg corrections supported the significant *p*-values from PERMANOVA. All pairwise tests comparing samples from station 6 or 7 with samples from station 1 to 5 displayed significant PERMDISP results. Likewise, pairwise tests involving the particle-attached water fraction (“3”) displayed significant or near-significant PERMDISP results.

**Table 1:** Results of global and pairwise PERMANOVA, Benjamini-Hochberg correction, and corresponding PERMDISP of microbial communities from the different sample sources PE, PS, wood (L), particle-attached (3), and free-living water fraction (0.2).

	**Groups**	**PERMANOVA**		**perms**	**p(BH)**	**PERMDISP**
		***t***	***p***			***p***
Global			**0.001**	995		0.148
Pairwise	PE, PS	0.733	0.816	999	0.816	0.726
	**PE, L**	1.826	**0.008**	999	**0.010**	0.794
	**PS,L**	1.783	**0.005**	998	**0.007**	0.584
	**PE,3**	2.033	**0.002**	999	**0.003**	**0.015**
	**PS,3**	2.072	**0.001**	998	**0.003**	**0.015**
	**L, 3**	2.028	**0.002**	999	**0.003**	**0.034**
	**PE,0.2**	2.642	**0.001**	998	**0.003**	0.925
	**PS,0.2**	2.706	**0.001**	999	**0.003**	0.687
	**L,0.2**	2.572	**0.001**	999	**0.003**	0.914
	**3,0.2**	1.573	**0.017**	998	**0.019**	0.061

*Analyses are based on 16S rRNA data from all 7 stations. Significant results (P < 0.05) are highlighted in bold. perms, permutations; BH, Benjamini-Hochberg correction*.

PCO of all samples displayed a gradient, which was formed by salinity, temperature and, negatively correlating, nutrient concentrations (Figure 2). The samples roughly line up along this gradient according to station. However, the first two axes solely amount to 38% of the variation within the communities. Samples from the coastal Baltic and Warnow form two joint clusters, one comprising the plastic and wood-associated communities, the other the water communities. All samples from the WWTP form another cluster.

**Figure 3:**
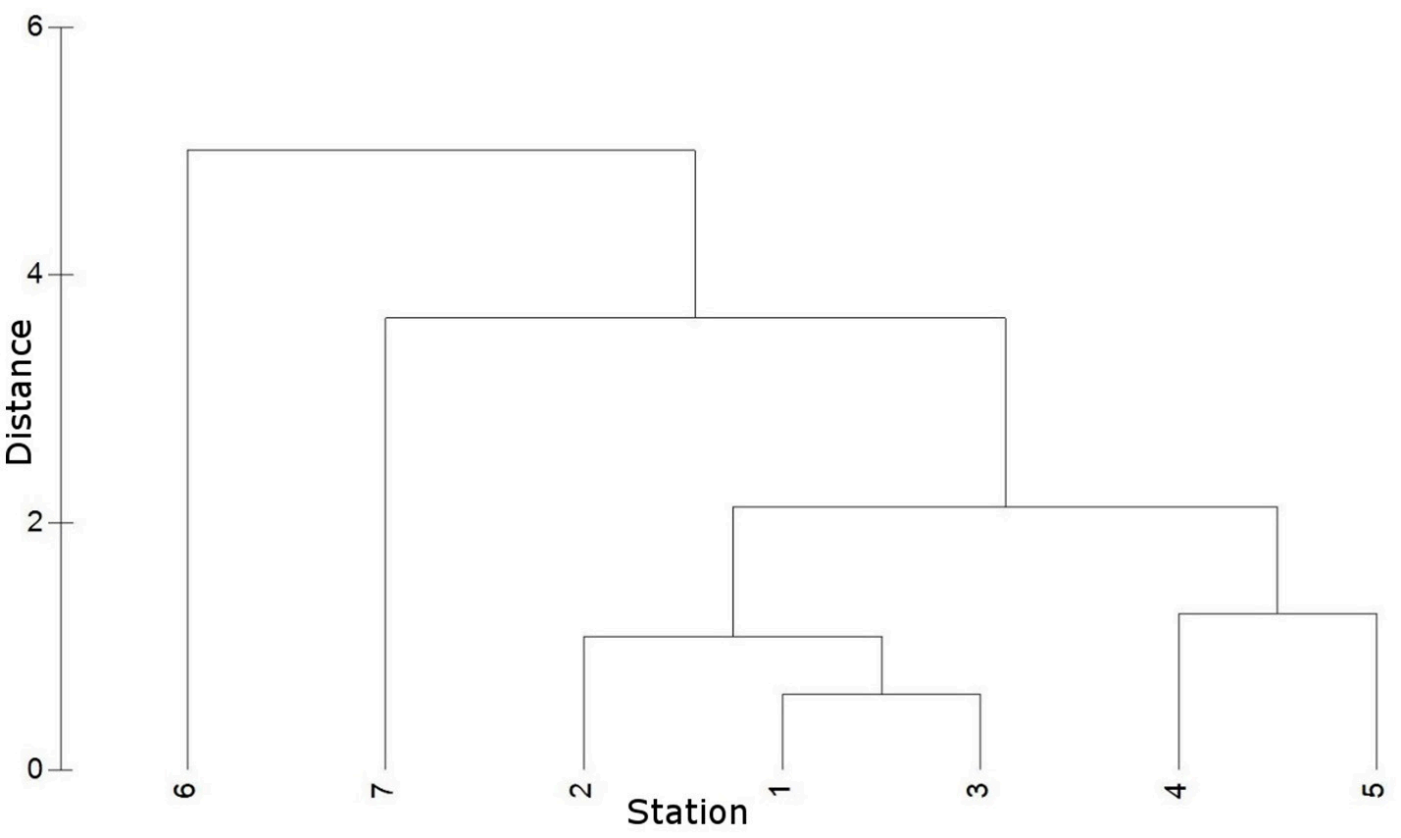
Hierarchical cluster (group average) based on Euclidian distances of environmental data (temperature, salinity, ${\text{PO}}_{4}^{3-}$, ${\text{NO}}_{3}^{-}$, ${\text{NO}}_{2}^{-}$, ${\text{NH}}_{4}^{+}$). Cluster displays similarity of environmental conditions at stations 1 to 7.

Hierarchical clustering of the environmental data indicated similar environmental conditions at stations 1 to 3 (coastal Baltic Sea) and at stations 4 and 5 (Warnow estuary) (Figure 3). Station 6 and 7 (WWTP) cluster away from the other stations, while displaying different conditions between each other. Station 1, 2, and 3 featured marine/brackish conditions (salinity 13.5–14.7) and lowest nutrient concentrations (e.g., 0.09–3.84 μmol ${\text{NO}}_{3}^{-}$/l) (Figure 1, Table 2). Station 4 and 5 displayed lower salinities (6.5–10.0, brackish conditions) and increased nutrient concentrations (e.g., 6.03–48.76 μmol ${\text{NO}}_{3}^{-}$/l). Station 6 and 7 had freshwater (salinity of < 0.5) and high nutrient levels (e.g., 701.80–751.35 μmol ${\text{NO}}_{3}^{-}$/l). The water temperature in the sampling period was similar at stations 1–5 (17.0–18.3°C), while lower at stations 6 and 7 (14.7–15.2°C).

**Table 2:** Temperature, salinity and nutrient concentrations (${\text{PO}}_{4}^{3-}$, ${\text{NO}}_{3}^{-}$, ${\text{NO}}_{2}^{-}$, ${\text{NH}}_{4}^{+}$, and SiO_2_) of the water at stations 1–7 at the start and end dates of the *in situ* experiment.

	**Station**						
	**1**	**2**	**3**	**4**	**5**	**6**	**7**
Start	19/08/14	20/08/14	20/08/14	19/08/14	19/08/14	28/04/15	28/04/15
End	03/09/14	04/09/14	04/09/14	03/09/14	03/09/14	12/05/15	12/05/15
T [°C]	17.40	18.30	17.00	17.95	17.60	15.20	14.75
	0.50	0.60	0.50	0.55	0.10	0.90	0.95
S	14.70	13.45	13.45	10.05	6.55	0.00	0.00
	3.70	2.95	3.15	0.35	4.25	0.00	0.00
${\text{PO}}_{4}^{3-}$ [μmol/l]	0.322	1.230	0.819	2.143	3.310	0.351	2.032
	0.106	0.188	0.133	0.291	1.117	0.051	0.400
${\text{NO}}_{3}^{-}$ [μmol/l]	0.090	2.666	3.841	48.757	6.032	751.350	701.804
	0.000	1.406	3.330	35.068	0.591	170.728	125.371
${\text{NO}}_{2}^{-}$ [μmol/l]	0.041	0.347	0.169	0.593	0.703	11.417	0.454
	0.011	0.185	0.045	0.044	0.380	7.699	0.235
${\text{NH}}_{4}^{+}$ [μmol/l]	0.432	3.377	2.283	7.554	5.471	24.949	1.231
	0.069	0.581	0.199	1.334	4.287	21.766	0.705
SiO_2_ [μmol/l]	5.191	17.838	11.148	146.962	>40	155.154	148.929
	0.649	5.629	3.076	9.885	na	31.888	19.942

*Numbers are mean values with standard deviation below. S, Salinity; T, Temperature*.

**Figure 4:**
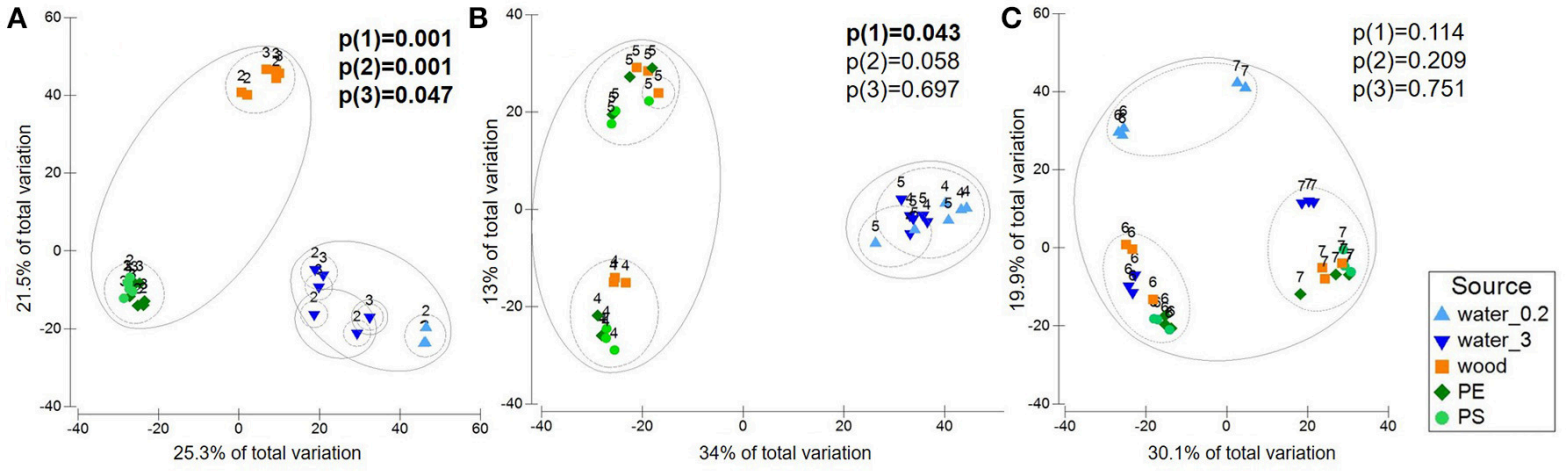
PCO displaying variation in microbial community composition in the different sample sources water (free-living fraction=water_0.2; particle-attached fraction=water_3), wood, PE, PS. Labels refer to sampling stations. Circles indicate 20 (straight line) and 40% (dashed line) of similarity. Plots are based on Bray-Curtis similarity matrices of square root-transformed relative abundances of OTUs (16S rRNA gene data), displaying the “Baltic” **(A)**, the “Warnow” **(B)**, the “WWTP” **(C)** dataset. Also given are *P*-values of pairwise PERMANOVA (Monte Carlo) from the tests (1) wood vs. PE, (2) wood vs. PS, (3) PE vs. PS, with significant values (*p* < 0.05) in bold.

Based on the clustering of the environmental data, the sequencing data were partitioned into “Baltic” (station 2 and 3), “Warnow” (station 4 and 5), and “WWTP” (station 6 and 7) datasets. Pairwise PERMANOVA (Monte Carlo) revealed a significant separation between all sample sources in the “Baltic” dataset (Additional file 3). This includes the communities attached to plastic compared to wood (*p* = 0.001) as well as the communities colonizing PE compared to PS (*p* = 0.047). The “Warnow” dataset displayed no significant separation between the communities attached to PE compared to PS, and to wood compared to PS. All other sample sources, however, differed significantly. In the “WWTP” dataset, no significant differences were found between either of the attached communities. Solely the free-living and attached communities differed significantly. The different levels of substrate specificity of the microbial communities in the three datasets are visualized in Figure 4. All significant PERMANOVA results were supported by Benjamini-Hochberg corrections, except the differentiation between communities associated with wood and PE in the “Warnow” dataset. Dispersion effects could be detected in some cases (Additional file 3).

**Figure 5:**
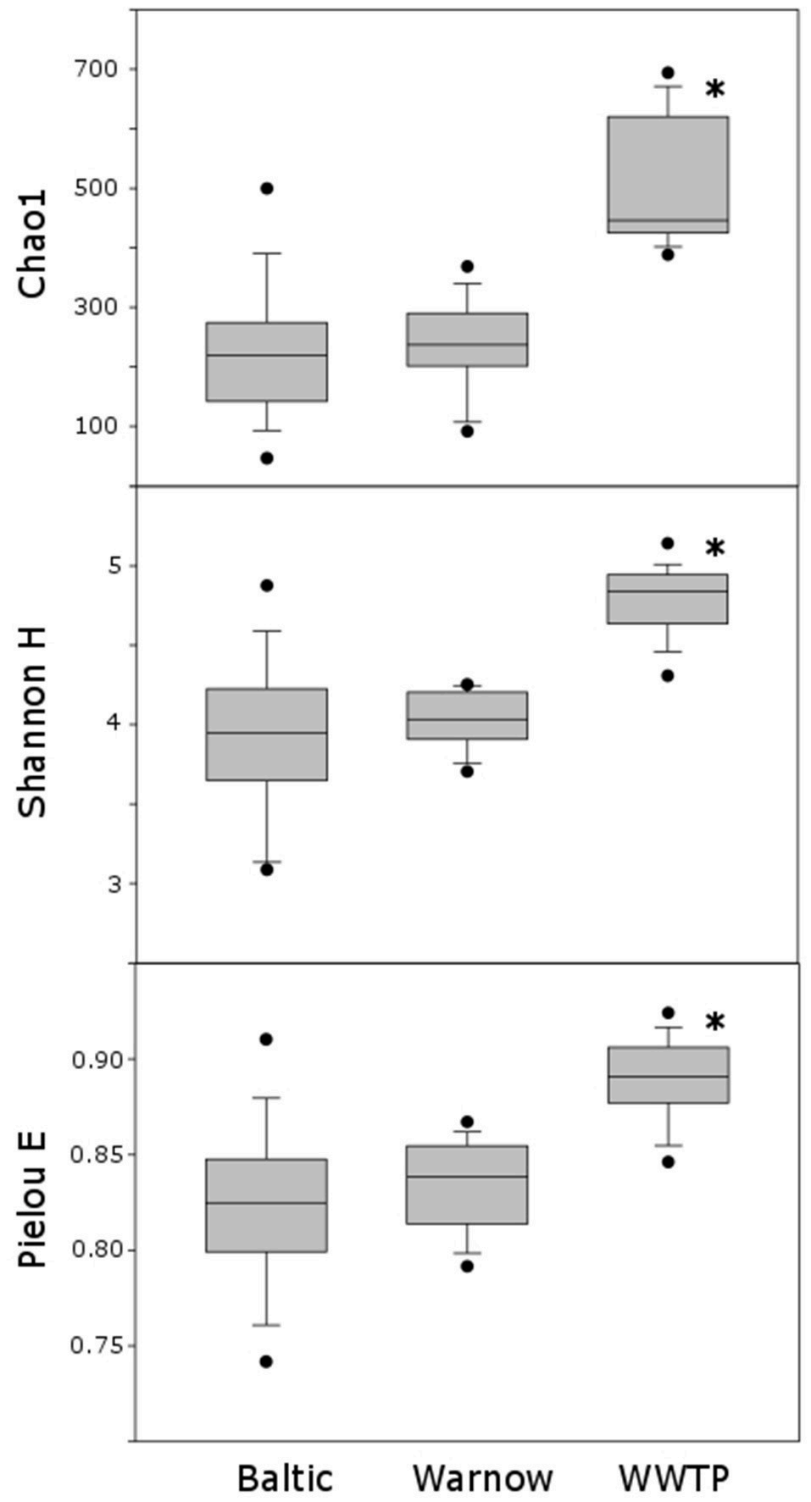
Box plots illustrating the Chao1, Shannon H and Pielou E indices of datasets “Baltic,” “Warnow,” “WWTP”; ^*^indicates significant differences (*p* < 0.01) between datasets.

Based on alpha diversity analyses, assemblages from the “WWTP” dataset displayed significantly higher richness, diversity and evenness compared to the other stations (Figure 5).

### Partitioning of MP-associated microbial communities

**Figure 6:**
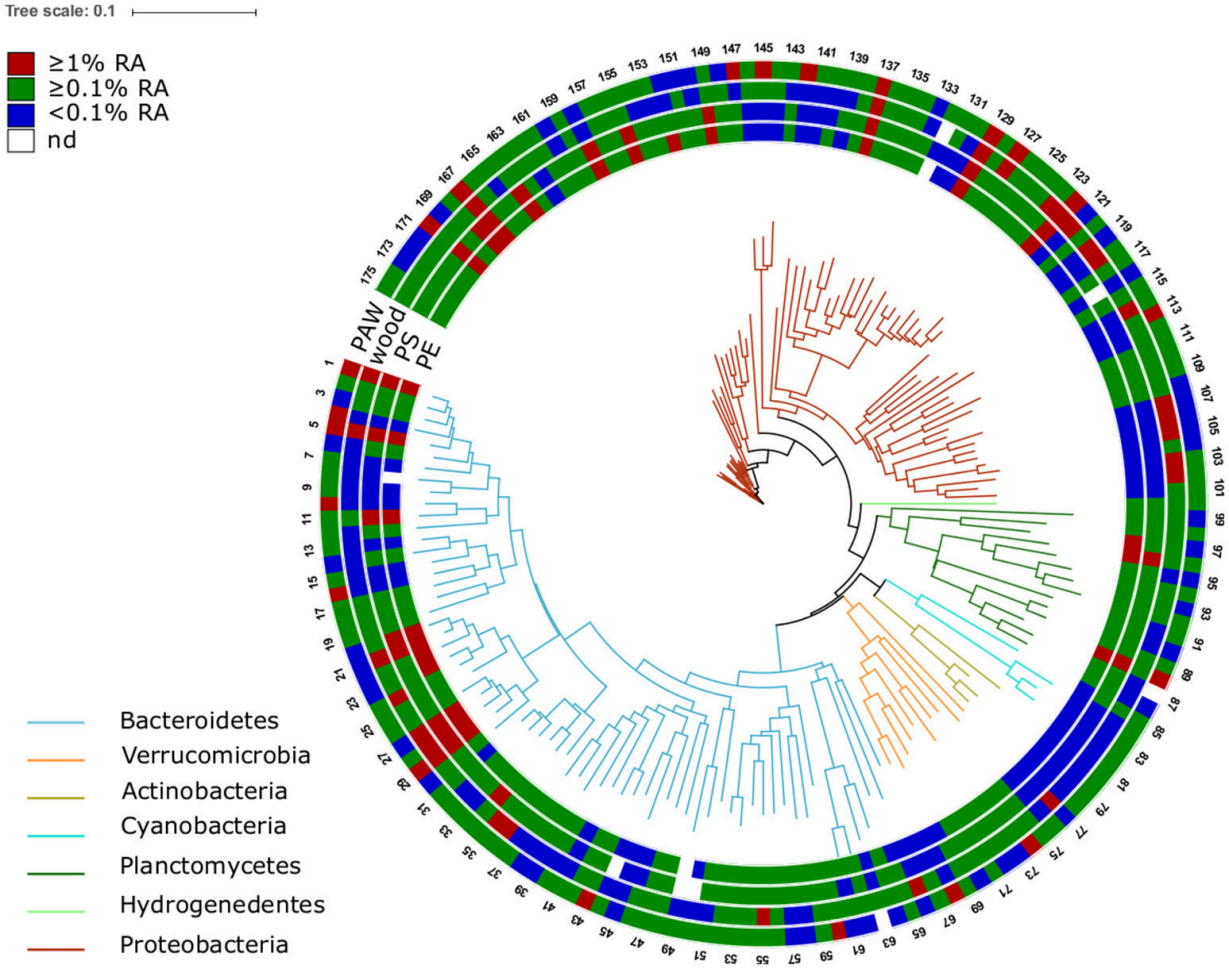
Phylogenetic tree of the 175 OTUs (16S rRNA data based) displaying an average relative abundance of ≥0.1% across the whole dataset. The colors of the branches refer to different bacterial phyla. The outer rings represent different sample sources: particle-attached water community (PAW), wood-, PE-, and PS-associated assemblages. The colors in the rings indicate the mean relative abundance of the OTUs in the individual sample sources across all stations. Scale bar represents 10 nucleotide substitutions per 100 nucleotides. Families with ≥ 3 OTUs are listed below. For more taxonomic details of OTUs see Additional file 4. 1–36: *Flavobacteriaceae*; 41–45: *Cryomorphaceae*; 52–58, 60: *Saprospiraceae*; 68, 69, 71, 73–75: *Verrucomicrobiaceae*; 78–80: *Microbacteriaceae*; 82–84: Cyanobacteria Family I; 86–96: *Planctomycetaceae*; 106, 107, 118, 119: *Cellvibrionaceae*; 122–130: *Comamonadaceae*; 131, 134–136: *Rhodocyclaceae*; 144–146: *Campylobacteraceae*; 150, 152, 153: *Erythrobacteraceae*; 155–158: *Hyphomonadaceae*; 159–175: *Rhodobacteraceae*.

Some families displayed high relative abundances at all stations in the PE and PS-associated as well as other communities: *Flavobacteriaceae* (esp. the genus *Flavobacterium*), *Rhodobacteraceae, Methylophilaceae* (*Methylotenera*), and *Planctomycetaceae* (*Planctomyces, Pirellula*; decreasing from station 1 to 7) (Figure 6 Additional file 4). The family *Hyphomonadaceae* (Figure 6 OTU 155-158), in particular the genus *Hyphomonas*, was found abundantly at stations 1–4 and 7, being consistently more abundant on both plastic surfaces (up to 6.3% relative abundance per sample) than on natural surfaces. Within the family *Planctomycetaceae, one* OTU assigned to the genus *Blastopirellula* was very abundant solely on plastic (up to 6.8%; Figure 6 OTU 96). At the Baltic and Warnow stations, the family *Erythrobacteraceae* (genus *Erythrobacter*, Figure 6 OTU 152) was abundant on PS and particularly on PE (up to 4.1%). One OTU (Figure 6 OTU 149), classified as *Sphingopyxis* (*Sphingomonadaceae*), displayed high relative abundances solely in the plastic-assemblages, particularly at station 7 (up to 8%).

LEfSe analysis (considering solely OTUs with a mean relative abundance ≥0.1%) confirmed the significant differential abundance in the plastic-assemblages of the above described genera *Hyphomonas, Erythrobacter, Blastopirellula*, and *Sphingopyxis* (Additional file 5). Other taxa were also significantly more abundant on plastic, despite not showing overall high relative abundances. These include the family *Phyllobacteriaceae* and the genus *Tenacibaculum* (*Flavobacteriaceae*). Likewise, the communities were tested toward their preference for PE or PS (Additional file 6). Most differential taxa between polymers were found in the “Baltic” (*N* = 37) dataset, and more differential abundant taxa were detected for PS- (*N* = 34) than PE- (*N* = 12) assemblages. Members of the families *Erythrobacteraceae* were in higher abundances associated with PE, while members of the families *Verrucomicrobiaceae* were significantly more abundant on PS.

### Occurrence of MP-associated potentially pathogenic bacteria

No increased relative abundances of potential pathogens on plastic compared to other sample types were detected. The genus *Vibrio* (*Vibrionaceae*) was found in plastic-attached communities, but LEfSe revealed significant higher abundance of this genus in wood-associated samples (LDA score = 4.7). The 16S rRNA gene barcode data were supported by qPCR. Of 65 samples tested toward the occurrence of *Vibrio* sp., 27 gave positive results, with members of the genus *Vibrio* being found at all tested stations and in all tested sources (Additional file 7). The particle-attached water communities displayed the most positive *Vibrio* sp. samples (*N* = 10), while the highest *Vibrio* abundances were detected for wood samples (1.30^*^10^7^-2.16^*^10^8^ gene copies/ng DNA, station 2). Solely two positive samples each were found for the plastic assemblages, with *Vibrio* concentrations of up to 6.95^*^10^4^ copies/ng at PS at station 4. Wood and water communities from station 4, however, displayed higher *Vibrio* abundances (1.16–3.28^*^10^5^ copies/ng).

Some OTUs from the plastic-associated sequences were assigned to members of the family *Enterobacteriaceae*. The highest relative abundance of *Enterobacteriaceae* in plastic samples was found on PS at station 4 and 6 (0.13 and 0.12%), with higher abundances of this family in water samples at the same stations (up to 1.7%).

## Discussion

### MP as specific vector relevant under certain environmental conditions

The present study sheds light on the relevance of MP, in comparison with natural particles, as a vector for specific bacterial communities under different environmental conditions. Our field experiment revealed that plastic-associated assemblages show spatial variation, in accordance with previous studies (Hoellein et al., 2014; Oberbeckmann et al., 2014; Amaral-Zettler et al., 2015). Here, we now demonstrate that we can find a MP-specific microbiome in marine waters, after McCormick and coauthors established this for freshwater habitats already in 2014. Our study reveals for the first time that the degree of specificity depends on the ambient environmental conditions, and that we can refer to the term “plastisphere” (Zettler et al., 2013) solely in certain environmental scenarios. The scenario “lower nutrient levels, higher salinity, coastal system” led to substrate specificity in our study. In areas with lower nutrient concentrations (Baltic stations) we could not only observe a complete differentiation between plastic, wood and particle-attached water communities but also significant differences between the PE- and PS-assemblages. At the stations with higher nutrient concentrations, especially within the WWTP, we could not detect major differences between the communities associated with different substrates. The fungal community from the same experimental setup showed similar patterns, as published recently by Kettner et al. (2017). We detected, using PERMDISP analyses, dispersion factors to play a role in the differentiation between some of the sample groups. Linear discriminant analysis and visualization via PCO, however, support that location factors play at least an equivalent role.

Rummel et al. (2017) summarized substrate parameters relevant for microbial colonization, e.g., surface roughness and hydrophobicity. Considering the significant differences between PE, PS, and wood biofilms at the coastal stations, these factors assumingly did play a role. The surface parameters, however, were overall secondary to the environmental influence. Potentially, surface parameters play mainly a role in early colonization stages, with spatial factors increasing in importance over time. Alternatively, environmental factors have a stronger influence on shaping MP assemblages starting with the initial attachment, e.g., by determining the conditioning film. In order to clarify these early attachment processes, future experiments on MP biofilms might include the colonization phase of the first few hours to days. In all aquatic systems biofilm formation, architecture and stability depend on complex interactions with biotic and abiotic factors (Gerbersdorf and Wieprecht, 2015). A study by De Tender et al. (2015) suggested an influence of factors as salinity, temperature and oxygen content, on microbial colonization of macroplastics. The affirmation of these complex influences on MP biofilms can now be provided with our data. Several parameters including nutrient concentrations, salinity, and temperature contributed to shaping the microbial communities on PE and PS.

Low nutrient levels trigger the attachment to surfaces in many bacterial species, while under high nutrient levels biofilm formation seems less advantageous (Stanley and Lazazzera, 2004). Zobell postulated already in 1943 that biofilm formation plays a major role in nutrient-low waters, and mentioned also plastic to be surface active (Zobell, 1943). Over 70 years later, we confirm his findings with regard to MP. Presumably, the microbial colonization at the coastal stations with lower nutrient availability was a directed process, specific to each surface type (e.g., hard vs. soft substratum), and involving microorganisms with a more specialized metabolism. The more nutrients that are available, the quicker a conditioning film and primary biofilm can develop, the faster a secondary, less substrate specific, biofilm can be established. Datta et al. (2016) investigated micro-scale successions on marine particles, and described three phases of colonization. Specifically, the third phase is dominated by secondary consumers and displays the highest biofilm diversity. The term “Fast-but-inefficient metabolism strategy” for bacteria was coined by Polz and Cordero (2016) based on a study by Roller et al. (2016): under high nutrient flux, ribosomal RNA operon numbers increase, followed by higher biomass production but lower carbon use efficiency. Valuable metabolic intermediates are excreted, which in a biofilm can be used by other colonizers. This way, an interactive biofilm network can develop, leading to high richness and diversity. In agreement with this, in our study bacterial richness and diversity was higher for the Warnow and especially the nutrient-rich WWTP compared to the Baltic stations.

MP is ubiquitously present in the marine ecosystem with particularly high concentrations in the subtropical ocean gyres (Law et al., 2010; Eriksen et al., 2013; Cózar et al., 2014). Our findings suggest that the significance of MP in these accumulation zones could be even higher than believed to date. Besides plastic, these waters harbor a rich microbial diversity, such as the oligotrophic Sargasso Sea (Venter et al., 2004). Leaving aside the issue of attached potential pathogenic or invasive microorganisms, merely the formation of non-natural, plastic-specific biofilms in such a plastic accumulation zone might have implications for the sensitive ecosystem. The microbial communities in the oceans form complex interactions with each other and with other organisms and play key roles in vital ecosystem processes such as primary production and nutrient cycling (Falkowski et al., 2008). Potentially, these natural synergies are being altered by MP biofilms under certain environmental conditions, including low nutrient availability.

### *Hyphomonadaceae* and *Erythrobacteraceae* are abundant specifically in MP biofilms

On the family level, PE and PS colonizing communities consisted of many typical aquatic colonizers, such as members of the family *Rhodobacteraceae* (Dang et al., 2008). Several families abundant on the plastic after exposure, have previously been found associated with various hard substrata, plastic surfaces just being one of them (Hoellein et al., 2014; Oberbeckmann et al., 2016). Our *in situ* experiment, however, identified several taxa attracted specifically by plastic surfaces, especially at the coastal Baltic stations. Two families were abundant exclusively on MP across several stations, namely *Hyphomonadaceae* (mainly *Hyphomonas*) and *Erythrobacteraceae* (mainly *Erythrobacter*). Zettler et al. (2013) found OTUs affiliated with these families to be abundantly associated with, some of them even unique to, plastic fragments floating in the Sargasso Sea. Plastic fragments in the North Pacific Gyre were abundantly colonized by members of the family *Hyphomonadaceae* and to a slightly lesser extent also *Erythrobacteraceae* (Bryant et al., 2016). Members of both families were also part of the “core microbiome,” defined by De Tender et al. (2017) for biofilms on PE sheets and ropes incubated on the seafloor in the Belgian part of the North Sea. This conformity with our findings indicates that the ecological conclusions from our Baltic Sea study apply to other ecosystems as well.

*Hyphomonadaceae* are prosthecate bacteria and produce the polysaccharide holdfast, which enables them to attach firmly to surfaces as primary colonizers. The prosthecae allow them to take up nutrients in a wide surrounding area (Abraham and Rohde, 2014). The outer membrane of *Hyphomonadaceae* features transport systems that actively take up compounds too large for passive diffusion. The efficient use of nutrients and the ability to attach firmly to even smooth surfaces makes members of the *Hyphomonadaceae* prime candidates to colonize MP.

*Erythrobacteraceae* on the other hand, stand out due to their production of carotenoids and bacteriochlorophyll *a* (Tonon et al., 2014), which is beneficial for a life on surface-floating plastic debris. Interestingly, this family has a high potential for biotechnological purposes, due to the possession of important hydrolases. There is very strong evidence for the ability of members of the genus *Erythrobacter* to degrade PAH (Gao et al., 2015; Zhuang et al., 2015). Members of *Erythrobacter* might be able to degrade PAH associated with plastic. MP sorb PAH from the surrounding water, and especially PE has a high capacity to accumulate PAH (Adams et al., 2007; Teuten et al., 2009; Rochman et al., 2013). Therefore, the metabolization of MP-associated PAH by members of *Erythrobacter* is a likely explanation for the significantly higher abundance of *Erythrobacter* on PS and particularly PE compared to natural surfaces in our study.

The genus *Tenacibaculum* (*Flavobacteriaceae*) has consistently been reported to be abundant in plastic colonizing communities, across polymers, and sampling areas (Zettler et al., 2013; Bryant et al., 2016; Oberbeckmann et al., 2016; De Tender et al., 2017). While not being among the most abundant taxa on MP from our experiment, *Tenacibaculum* displayed significantly higher abundance on PE and PS than in other sample types, and should be investigated further regarding its role in MP biofilms.

### Colonization of PE and PS by potentially pathogenic microorganisms

Potentially pathogenic *Vibrio* spp. have been recently in the spotlight, following a study detecting their high relative abundances (>20%) on a PP particle from the North Atlantic (Zettler et al., 2013). Subsequently, members of the genus *Vibrio* were also found on plastic debris at a Scottish beach (Quilliam et al., 2014) and on floating MP in the North and Baltic Sea (Kirstein et al., 2016). Bryant et al. (2016), however, did not confirm high abundances of *Vibrionaceae* on plastic debris from the North Pacific Gyre. Our study revealed that *Vibrio* bacteria did colonize PE and PS in the Warnow and coastal Baltic Sea, but in lower concentrations than they colonized the surface of wood and other natural particles in the water column. The enrichment of *Vibrio* sp. in aquatic environments is common on wooden debris, plants and other natural surfaces (Takemura et al., 2014), but might play a minor role with regard to the colonization of MP.

It has also been previously hypothesized that MP in WWTPs might take up sewage-related microorganisms and import them into the environment via the treated wastewater effluent (McCormick et al., 2014; Oberbeckmann et al., 2015). Our station 4, located in the Warnow estuary close to a WWTP runoff, featured very high nutrient levels, indicating a strong impact by the treated wastewater. Indeed, elevated levels of the family *Enterobacteriaceae* were detected on PE and PS at station 4 as well as within the WWTP (station 7). This family comprises several potentially gut-related and pathogenic genera (Octavia and Lan, 2014). The relative abundances of *Enterobacteriaceae* on MP, however, remained low (<0.5%) in relation to the levels in the water communities outside and within the WWTP. Despite the low numbers of potential pathogens associated with MP, the possible long-term effect of MP colonization should be considered. Plastic fragments represent more stable surfaces than wood or most other natural particles, which might lead to more durable biofilms. Already Hoellein et al. (2014) presumed that plastic biofilms might stay intact longer and transport biofilms further compared to natural surfaces.

One genus significantly more abundant on plastic than in other sample types within the WWTP was *Sphingopyxis* (*Sphingomonadaceae*). *Sphingomonadaceae* are known to be a reservoir for antibiotic-resistance (Vaz-Moreira et al., 2011; Iredell et al., 2016). More specifically, the genus *Sphingopyxis* has been described as secondary colonizer of PE drinking water pipes (Douterelo et al., 2014), and a draft genome analysis revealed the presence of a class I integron associated with two antibiotic resistance genes (Gomez-Alvarez et al., 2016). Class I integrons are often embedded in plasmids or transposons, enabling their lateral transfer (Gillings et al., 2008), and have been suggested to be a proxy for anthropogenic pollution (Gillings et al., 2015). The potential for MP to be a hotspot for the transport and transfer of antibiotic resistance has not been investigated so far, but there is urgent need to do so.

## Conclusions

Our study highlights the role of MP for specific microbial communities as subject to environmental conditions, including nutrient levels. Consequently, this emphasizes the relevance of MP accumulation zones, such as the oligotrophic Sargasso Sea. Most potentially pathogenic taxa were not enriched on MP; however, the presence of certain bacteria commonly associated with antibiotic resistance on MP in a WWTP suggests that small plastic particles may be hotspots for horizontal gene transfer. In comparison with natural surfaces, MP attracted significantly higher numbers of members of the families *Hyphomonadaceae* and *Erythrobacteraceae*, with the latter potentially being associated with PAH degradation. Future studies should examine, whether our short-term observations can be extended to longer time frames and to other important marine areas.

## Author contributions

SO and ML designed the experiment and analyzed the data. SO and BK performed the experiments and measurements. All authors were involved in data interpretation, drafting of the manuscript, and read and approved the final manuscript.

### Conflict of interest statement

The authors declare that the research was conducted in the absence of any commercial or financial relationships that could be construed as a potential conflict of interest.
