# Supplementary material for: Environmental Factors Support the Formation of Specific Bacterial Assemblages on Microplastics
Source: Front Microbiol. 2018 Jan 19;8:2709. doi: 10.3389/fmicb.2017.02709 (PMC5785724; doi:10.3389/fmicb.2017.02709)
Supplement: Supplementary file 1 [file DataSheet1.docx]

ADDITIONAL FILES 1 to 7

**Additional file 1: Supplemental information on Material & Methods**

*DNA isolation*

Pellets and filter were transferred to a 2 ml tube containing 600 µl SLS buffer (100 ml contain 12.3 ml SLS [20 % w/v], 3 ml 0.5 M Na-Acetate [pH 7.5], 84.7 ml ddH2O). Five of each 2 and 3 mm zirconium beads were added to the pellets or filters, respectively. Bead beating took place for 2 min at 2,000 rpm, followed by a short break and an additional beating for 1 min. Tubes were centrifuged (14,000 rpm, 2 min) and the supernatant transferred to a new tube. The procedure of bead beating was repeated as described above, therefore 400 µl SLS buffer was added to the pellets / filter and beads. The supernatants were merged and 1 vol. phenol/chloroform was added. Tubes were centrifuged (14,000 rpm, 5 min), the top phase containing extracted DNA was transferred to a fresh tube and 1 vol. chloroform added. The content was mixed well and centrifuged (14,000 rpm, 5 min). The top phase was transferred to a fresh tube and 1.5 vol. precipitation mix (100 ml contain 96 ml ethanol [abs.], 4 ml Na-Acetate [3 M]) was added, and then stored overnight at -20 C. DNA was then pelleted by centrifugation (4°C, 14,000 rpm, 40 min), supernatant discarded, pellet washed with ice-cold 70% EtOH (500 µl), centrifuged (14,000 g, 15 min), and supernatant carefully removed. This was followed by an additional ethanol washing step (as described above). The pellet was dried and resuspended in sterile nuclease-free water (30 µl). DNA extraction yields were measured with a NanoDrop Spectrophotometer (Kisker, DE).

*Amplification and library generation*

For the nested-PCR, the primer pair 27F and 1492R was used with each PCR tube containing 3 µl 5x PCR buffer (Herculase, Agilent Technologies, USA), 9.725 µl PCR grade water, 1 µl template DNA, 0.3 µl BSA [20 mg/ml] (Thermo Fisher Scientific, USA), 0.15 µl dNTP mix [100 µmol/L], 0.075 µl Pfu (Herculase, Agilent Technologies, USA) and 0.375 μl of each primer [10 µmol/L]. PCR grade water served as negative control and DNA of a known bacterial strain as positive control. Thermal cycling (MyCyclerTM thermal, Thermo Fisher Scientific, USA) consisted of an initial denaturation of 94°C for 5 min, followed by 16 cycles of 94°C for 45 sec, 45°C for 45 sec, and 72°C for 1.5 min, with a final extension at 72°C for 10 min. Subsequently, or directly, amplification of the V4 region was carried out with the primer pair 515F and 806R, both containing the Illumina overhang adapter sequences as outlined in the Illumina “16S Metagenomic Sequencing Library Preparation” protocol (www.illumina.com). 5 µl 5x PCR buffer was mixed with 1-2 µl template DNA, 0.5 µl BSA [20 mg/ml], 0.25 µl dNTP mix [100 µmol/L], 0.125 µl Pfu and 0.625 μl of each primer [10 µmol/L]. The mixture was filled up to 25 µl with PCR grade water. As described above, negative and positive controls were used. Thermal cycling consisted of an initial denaturation of 95°C for 2 min, followed by 25 cycles of 95°C for 20 sec, 55°C for 15 sec, and 72°C for 1 min, with a final extension at 72°C for 10 min.

Successful amplification and the correct amplicon size of 290 bp was confirmed by agarose gel electrophoresis (1.2 % w/v in 0.5x TBE buffer at 110 V) and imaging of ethidium bromide (Boehringer Mannheim, Stuttgart, DE)-stained DNA by UV light. PCR clean-up, index PCR, library quantification, normalization and pooling was performed according to the above referred manual. Bioanalyzer DNA 1000 chips (Agilent Technologies, USA) and Qubit kits (Thermo Fischer Scientific, USA) were used for quantity and quality controls of each individual sample and the final library pools. Ten percent PhiX control was spiked into each of the pools. 4 pM of each library pool was subjected to paired-end sequencing runs using 500 cycle V2 chemistry kits on an Illumina MiSeq machine. All raw data fastq files were recovered from the machine and used for further sequence data processing as outlined below.

*qPCR*

PCR conditions were the following: initial denaturation of 95°C for 10 min, followed by 40 cycles of 95°C for 10 sec, 60.2°C for 10 sec, 72°C for 5 sec, and 83°C for 10 sec. This was followed by one step at 72°C for 10 min, one step at 95°C for 1 min, and one step at 60°C for 30 sec. To generate the melting curve, a heating step gradient from 50 to 92°C was added at the end of the PCR run, with the temperature increasing by 0.5°C with each 10 sec step. 8.290 µl 2x KAPA SYBR FAST BioRad (peqlab) were mixed with 1 µl template DNA [100ng], and 0.4 μl of each primer [10 µmol/L]. The mixture was filled up to 15 µl with PCR grade water. PCR products of the standard DNA were purified using the Agencourt AMPure XP system (Beckman-Coulter, USA).

**Additional file 2**: Global and pairwise PERMANOVA of microbial assemblages from the 7 sampling stations (factor ‘environment’), as well as adjusted p after Benjamini-Hochberg correction and corresponding PERMDISP results. Analyses are based on 16S rRNA data from all sample sources (water, wood, PE, PS). Significant results (p< 0.05) are highlighted in bold.

|  |  | PERMANOVA | |  |  | PERMDISP |
| --- | --- | --- | --- | --- | --- | --- |
|  | Groups | t | p | perms | P(BH) | p |
| Global |  |  | **0.001** | 999 |  | **0.001** |
| Pairwise | **1, 4** | 2.230 | **0.001** | 998 | **0.001** | 0.803 |
|  | **1, 5** | 2.704 | **0.001** | 997 | **0.001** | 0.637 |
|  | **1, 3** | 1.531 | **0.013** | 998 | **0.014** | 0.556 |
|  | **1, 2** | 1.725 | **0.002** | 999 | **0.003** | 0.557 |
|  | **1, 6** | 4.902 | **0.001** | 998 | **0.001** | **0.003** |
|  | **1, 7** | 4.436 | **0.001** | 999 | **0.001** | **0.008** |
|  | **4, 5** | 1.647 | **0.008** | 999 | **0.009** | 0.761 |
|  | **4, 3** | 1.694 | **0.005** | 997 | **0.006** | 0.575 |
|  | **4, 2** | 1.556 | **0.024** | 998 | **0.025** | 0.612 |
|  | **4, 6** | 4.612 | **0.001** | 999 | **0.001** | **0.001** |
|  | **4, 7** | 4.212 | **0.001** | 997 | **0.001** | **0.001** |
|  | **5, 3** | 2.202 | **0.001** | 998 | **0.001** | 0.724 |
|  | **5, 2** | 2.121 | **0.001** | 997 | **0.001** | 0.789 |
|  | **5, 6** | 4.193 | **0.001** | 998 | **0.001** | **0.001** |
|  | **5, 7** | 3.854 | **0.001** | 999 | **0.001** | **0.001** |
|  | 3, 2 | 1.074 | 0.298 | 999 | 0.298 | 0.932 |
|  | **3, 6** | 4.446 | **0.001** | 999 | **0.001** | **0.001** |
|  | **3, 7** | 4.031 | **0.001** | 998 | **0.001** | **0.004** |
|  | **2, 6** | 4.549 | **0.001** | 999 | **0.001** | **0.001** |
|  | **2, 7** | 4.147 | **0.001** | 998 | **0.001** | **0.003** |
|  | **6, 7** | 3.293 | **0.001** | 999 | **0.001** | 0.124 |

perms: permutations; BH: Benjamini-Hochberg correction

**Additional file 3**: Pairwise PERMANOVA and corresponding PERMDISP results of microbial communities from the different sample sources PE, PS, wood (L), particle-attached water fraction (3), free-living water fraction (0.2). Also shown are adjusted Monte Carlo p values after Benjamini-Hochberg correction. Analyses are based on 16S rRNA data, divided into the three datasets ‘Baltic’, ‘Warnow’, ‘WWTP’. Significant Monte Carlo, Benjamini-Hochberg, and PERMDISP results (P < 0.05) are highlighted in bold.

|  |  | PERMANOVA | |  |  |  | PERMDISP |
| --- | --- | --- | --- | --- | --- | --- | --- |
| Dataset | Groups | t | p | p(MC) | perms | p(MC_BH) | p |
| **Baltic** | **PE, PS** | 1.472 | **0.016** | **0.047** | 396 | **0.047** | **0.004** |
|  | **0.2, PE** | 3.249 | **0.011** | **0.001** | 84 | **0.003** | 0.196 |
|  | **0.2, PS** | 3.746 | **0.014** | **0.003** | 84 | **0.004** | 0.883 |
|  | **0.2, 3** | 1.604 | **0.009** | **0.044** | 84 | **0.047** | **0.013** |
|  | **0.2, L** | 3.752 | **0.014** | **0.002** | 84 | **0.004** | 0.989 |
|  | **3, PE** | 2.177 | **0.002** | **0.003** | 403 | **0.004** | **0.001** |
|  | **3, PS** | 2.264 | **0.004** | **0.003** | 409 | **0.004** | **0.002** |
|  | **3, L** | 2.511 | **0.003** | **0.003** | 414 | **0.004** | **0.004** |
|  | **L, PE** | 3.526 | **0.007** | **0.001** | 412 | **0.003** | **0.002** |
|  | **L, PS** | 3.798 | **0.006** | **0.001** | 412 | **0.003** | 0.826 |
| **Warnow** | PE, PS | 0.786 | 0.755 | 0.697 | 417 | 0.697 | 0.790 |
|  | **0.2, PE** | 2.818 | **0.002** | **0.001** | 409 | **0.003** | 0.370 |
|  | **0.2, PS** | 2.839 | **0.004** | **0.002** | 403 | **0.003** | 0.466 |
|  | **0.2, 3** | 1.728 | **0.004** | **0.019** | 404 | **0.027** | 0.363 |
|  | **0.2, L** | 2.846 | **0.001** | **0.001** | 416 | **0.003** | 0.379 |
|  | **3, PE** | 2.840 | **0.002** | **0.001** | 405 | **0.003** | **0.003** |
|  | **3, PS** | 2.819 | **0.003** | **0.001** | 401 | **0.003** | **0.014** |
|  | **3, L** | 2.881 | **0.004** | **0.002** | 404 | **0.003** | **0.007** |
|  | **L, PE** | 1.522 | **0.013** | **0.043** | 405 | 0.054 | 0.870 |
|  | L, PS | 1.506 | 0.03 | 0.058 | 404 | 0.064 | 0.911 |
| **WWTP** | PE, PS | 0.715 | 0.786 | 0.751 | 410 | 0.751 | 0.458 |
|  | **0.2, PE** | 2.505 | **0.002** | **0.003** | 403 | **0.015** | 0.339 |
|  | **0.2, PS** | 2.389 | **0.004** | **0.002** | 410 | **0.015** | **0.013** |
|  | **0.2, 3** | 2.076 | **0.005** | **0.012** | 408 | **0.030** | **0.018** |
|  | **0.2, L** | 2.147 | **0.001** | **0.007** | 408 | **0.023** | **0.001** |
|  | 3, PE | 1.518 | 0.074 | 0.068 | 416 | 0.136 | 0.671 |
|  | 3, PS | 1.311 | 0.081 | 0.180 | 401 | 0.225 | 0.296 |
|  | 3, L | 1.359 | 0.092 | 0.134 | 411 | 0.191 | **0.004** |
|  | L, PE | 1.353 | 0.106 | 0.114 | 413 | 0.190 | 0.071 |
|  | L, PS | 1.208 | 0.105 | 0.209 | 423 | 0.232 | **0.008** |

perms: permutations; MC: Monte Carlo; BH: Benjamini-Hochberg correction

**Additional file 4**: Assigned taxonomy of the 175 OTUs, which display an average relative abundance of ≥0.1% across the whole dataset (16S rRNA based, cluster cutoff level=0.03), see also Fig. 4. Reference database was SILVA SSU 123.

| **OTU** | **Domain; Phylum; Class; Order; Family; Genus** |
| --- | --- |
| 1 | Bacteria;Bacteroidetes;Flavobacteriia;Flavobacteriales;Flavobacteriaceae;Gaetbulibacter |
| 2 | Bacteria;Bacteroidetes;Flavobacteriia;Flavobacteriales;Flavobacteriaceae;unclassified |
| 3 | Bacteria;Bacteroidetes;Flavobacteriia;Flavobacteriales;Flavobacteriaceae;Gaetbulibacter |
| 4 | Bacteria;Bacteroidetes;Flavobacteriia;Flavobacteriales;Flavobacteriaceae;Formosa |
| 5 | Bacteria;Bacteroidetes;Flavobacteriia;Flavobacteriales;Flavobacteriaceae;unclassified |
| 6 | Bacteria;Bacteroidetes;Flavobacteriia;Flavobacteriales;Flavobacteriaceae;Aquibacter |
| 7 | Bacteria;Bacteroidetes;Flavobacteriia;Flavobacteriales;Flavobacteriaceae;Aquibacter |
| 8 | Bacteria;Bacteroidetes;Flavobacteriia;Flavobacteriales;Flavobacteriaceae;NS5_marine_group |
| 9 | Bacteria;Bacteroidetes;Flavobacteriia;Flavobacteriales;Flavobacteriaceae;NS5_marine_group |
| 10 | Bacteria;Bacteroidetes;Flavobacteriia;Flavobacteriales;Flavobacteriaceae;unclassified |
| 11 | Bacteria;Bacteroidetes;Flavobacteriia;Flavobacteriales;Flavobacteriaceae;unclassified |
| 12 | Bacteria;Bacteroidetes;Flavobacteriia;Flavobacteriales;Flavobacteriaceae;Dokdonia |
| 13 | Bacteria;Bacteroidetes;Flavobacteriia;Flavobacteriales;Flavobacteriaceae;Ulvibacter |
| 14 | Bacteria;Bacteroidetes;Flavobacteriia;Flavobacteriales;Flavobacteriaceae;unclassified |
| 15 | Bacteria;Bacteroidetes;Flavobacteriia;Flavobacteriales;Flavobacteriaceae;unclassified |
| 16 | Bacteria;Bacteroidetes;Flavobacteriia;Flavobacteriales;Flavobacteriaceae;NS3a_marine_group |
| 17 | Bacteria;Bacteroidetes;Flavobacteriia;Flavobacteriales;Flavobacteriaceae;Cellulophaga |
| 18 | Bacteria;Bacteroidetes;Flavobacteriia;Flavobacteriales;Flavobacteriaceae;unclassified |
| 19 | Bacteria;Bacteroidetes;Flavobacteriia;Flavobacteriales;Flavobacteriaceae;Robiginitalea |
| 20 | Bacteria;Bacteroidetes;Flavobacteriia;Flavobacteriales;Flavobacteriaceae;Flavobacterium |
| 21 | Bacteria;Bacteroidetes;Flavobacteriia;Flavobacteriales;Flavobacteriaceae;Flavobacterium |
| 22 | Bacteria;Bacteroidetes;Flavobacteriia;Flavobacteriales;Flavobacteriaceae;Flavobacterium |
| 23 | Bacteria;Bacteroidetes;Flavobacteriia;Flavobacteriales;Flavobacteriaceae;Flavobacterium |
| 24 | Bacteria;Bacteroidetes;Flavobacteriia;Flavobacteriales;Flavobacteriaceae;Flavobacterium |
| 25 | Bacteria;Bacteroidetes;Flavobacteriia;Flavobacteriales;Flavobacteriaceae;Flavobacterium |
| 26 | Bacteria;Bacteroidetes;Flavobacteriia;Flavobacteriales;Flavobacteriaceae;Flavobacterium |
| 27 | Bacteria;Bacteroidetes;Flavobacteriia;Flavobacteriales;Flavobacteriaceae;Flavobacterium |
| 28 | Bacteria;Bacteroidetes;Flavobacteriia;Flavobacteriales;Flavobacteriaceae;Flavobacterium |
| 29 | Bacteria;Bacteroidetes;Flavobacteriia;Flavobacteriales;Flavobacteriaceae;Flavobacterium |
| 30 | Bacteria;Bacteroidetes;Flavobacteriia;Flavobacteriales;Flavobacteriaceae;Flavobacterium |
| 31 | Bacteria;Bacteroidetes;Flavobacteriia;Flavobacteriales;Flavobacteriaceae;Polaribacter |
| 32 | Bacteria;Bacteroidetes;Flavobacteriia;Flavobacteriales;Flavobacteriaceae;Tenacibaculum |
| 33 | Bacteria;Bacteroidetes;Flavobacteriia;Flavobacteriales;Flavobacteriaceae;Tenacibaculum |
| 34 | Bacteria;Bacteroidetes;Flavobacteriia;Flavobacteriales;Flavobacteriaceae;unclassified |
| 35 | Bacteria;Bacteroidetes;Flavobacteriia;Flavobacteriales;Flavobacteriaceae;Actibacter |
| 36 | Bacteria;Bacteroidetes;Flavobacteriia;Flavobacteriales;Flavobacteriaceae;Lutibacter |
| 37 | Bacteria;Bacteroidetes;Flavobacteriia;Flavobacteriales;unclassified;unclassified |
| 38 | Bacteria;Bacteroidetes;Flavobacteriia;Flavobacteriales;unclassified;unclassified |
| 39 | Bacteria;Bacteroidetes;Flavobacteriia;Flavobacteriales;unclassified;unclassified |
| 40 | Bacteria;Bacteroidetes;Flavobacteriia;Flavobacteriales;Schleiferiaceae;Schleiferia |
| 41 | Bacteria;Bacteroidetes;Flavobacteriia;Flavobacteriales;Cryomorphaceae;Crocinitomix |
| 42 | Bacteria;Bacteroidetes;Flavobacteriia;Flavobacteriales;Cryomorphaceae;Fluviicola |
| 43 | Bacteria;Bacteroidetes;Flavobacteriia;Flavobacteriales;Cryomorphaceae;Salinirepens |
| 44 | Bacteria;Bacteroidetes;Flavobacteriia;Flavobacteriales;Cryomorphaceae;Owenweeksia |
| 45 | Bacteria;Bacteroidetes;Flavobacteriia;Flavobacteriales;Cryomorphaceae;Owenweeksia |
| 46 | Bacteria;Bacteroidetes;Bacteroidia;Bacteroidales;Marinilabiaceae;Marinifilum |
| 47 | Bacteria;Bacteroidetes;Sphingobacteriia;Sphingobacteriales;AKYH767;unclassified |
| 48 | Bacteria;Bacteroidetes;Bacteroidia;Bacteroidia_Incertae_Sedis;Draconibacteriaceae;unclassified |
| 49 | Bacteria;Bacteroidetes;Sphingobacteriia;Sphingobacteriales;NS11-12_marine_group;unclassified |
| 50 | Bacteria;Bacteroidetes;Sphingobacteriia;Sphingobacteriales;NS11-12_marine_group;unclassified |
| 51 | Bacteria;Bacteroidetes;unclassified;unclassified;unclassified;unclassified |
| 52 | Bacteria;Bacteroidetes;Sphingobacteriia;Sphingobacteriales;Saprospiraceae;unclassified |
| 53 | Bacteria;Bacteroidetes;Sphingobacteriia;Sphingobacteriales;Saprospiraceae;unclassified |
| 54 | Bacteria;Bacteroidetes;Sphingobacteriia;Sphingobacteriales;Saprospiraceae;unclassified |
| 55 | Bacteria;Bacteroidetes;Sphingobacteriia;Sphingobacteriales;Saprospiraceae;unclassified |
| 56 | Bacteria;Bacteroidetes;Sphingobacteriia;Sphingobacteriales;Saprospiraceae;unclassified |
| 57 | Bacteria;Bacteroidetes;Sphingobacteriia;Sphingobacteriales;Saprospiraceae;unclassified |
| 58 | Bacteria;Bacteroidetes;Sphingobacteriia;Sphingobacteriales;Saprospiraceae;unclassified |
| 59 | Bacteria;Bacteroidetes;Sphingobacteriia;Sphingobacteriales;Chitinophagaceae;Terrimonas |
| 60 | Bacteria;Bacteroidetes;Sphingobacteriia;Sphingobacteriales;Saprospiraceae;Lewinella |
| 61 | Bacteria;Bacteroidetes;Cytophagia;Cytophagales;Cytophagaceae;Leadbetterella |
| 62 | Bacteria;Bacteroidetes;Cytophagia;Cytophagales;Cytophagaceae;Leadbetterella |
| 63 | Bacteria;Bacteroidetes;unclassified;unclassified;unclassified;unclassified |
| 64 | Bacteria;Bacteroidetes;unclassified;unclassified;unclassified;unclassified |
| 65 | Bacteria;Bacteroidetes;Cytophagia;Cytophagales;Cyclobacteriaceae;Algoriphagus |
| 66 | Bacteria;Bacteroidetes;Bacteroidia;Bacteroidales;Bacteroidaceae;Bacteroides |
| 67 | Bacteria;Bacteroidetes;Cytophagia;Cytophagales;Flammeovirgaceae;Reichenbachiella |
| 68 | Bacteria;Verrucomicrobia;Verrucomicrobiae;Verrucomicrobiales;Verrucomicrobiaceae;Haloferula |
| 69 | Bacteria;Verrucomicrobia;Verrucomicrobiae;Verrucomicrobiales;Verrucomicrobiaceae;Luteolibacter |
| 70 | Bacteria;Verrucomicrobia;Verrucomicrobiae;Verrucomicrobiales;DEV007;unclassified |
| 71 | Bacteria;Verrucomicrobia;Verrucomicrobiae;Verrucomicrobiales;Verrucomicrobiaceae;unclassified |
| 72 | Bacteria;Verrucomicrobia;Verrucomicrobiae;Verrucomicrobiales;DEV007;unclassified |
| 73 | Bacteria;Verrucomicrobia;Verrucomicrobiae;Verrucomicrobiales;Verrucomicrobiaceae;unclassified |
| 74 | Bacteria;Verrucomicrobia;Verrucomicrobiae;Verrucomicrobiales;Verrucomicrobiaceae;Prosthecobacter |
| 75 | Bacteria;Verrucomicrobia;Verrucomicrobiae;Verrucomicrobiales;Verrucomicrobiaceae;Roseibacillus |
| 76 | Bacteria;Verrucomicrobia;Spartobacteria;Chthoniobacterales;FukuN18_freshwater_group;unclassified |
| 77 | Bacteria;Verrucomicrobia;Opitutae;Puniceicoccales;Puniceicoccaceae;Pelagicoccus |
| 78 | Bacteria;Actinobacteria;Actinobacteria;Micrococcales;Microbacteriaceae;unclassified |
| 79 | Bacteria;Actinobacteria;Actinobacteria;Micrococcales;Microbacteriaceae;Candidatus_Aquiluna |
| 80 | Bacteria;Actinobacteria;Actinobacteria;Micrococcales;Microbacteriaceae;ML602J-51 |
| 81 | Bacteria;Actinobacteria;Actinobacteria;Corynebacteriales;Mycobacteriaceae;Mycobacterium |
| 82 | Bacteria;Cyanobacteria;Cyanobacteria;SubsectionI;FamilyI;Synechococcus |
| 83 | Bacteria;Cyanobacteria;Cyanobacteria;SubsectionI;FamilyI;Synechococcus |
| 84 | Bacteria;Cyanobacteria;Cyanobacteria;SubsectionI;FamilyI;Synechococcus |
| 85 | Bacteria;Cyanobacteria;Melainabacteria;Obscuribacterales;unclassified;unclassified |
| 86 | Bacteria;Planctomycetes;Planctomycetacia;Planctomycetales;Planctomycetaceae;Planctomyces |
| 87 | Bacteria;Planctomycetes;Planctomycetacia;Planctomycetales;Planctomycetaceae;Planctomyces |
| 88 | Bacteria;Planctomycetes;Planctomycetacia;Planctomycetales;Planctomycetaceae;Planctomyces |
| 89 | Bacteria;Planctomycetes;Planctomycetacia;Planctomycetales;Planctomycetaceae;Planctomyces |
| 90 | Bacteria;Planctomycetes;Planctomycetacia;Planctomycetales;Planctomycetaceae;Planctomyces |
| 91 | Bacteria;Planctomycetes;Planctomycetacia;Planctomycetales;Planctomycetaceae;Planctomyces |
| 92 | Bacteria;Planctomycetes;Planctomycetacia;Planctomycetales;Planctomycetaceae;Pirellula |
| 93 | Bacteria;Planctomycetes;Planctomycetacia;Planctomycetales;Planctomycetaceae;Pirellula |
| 94 | Bacteria;Planctomycetes;Planctomycetacia;Planctomycetales;Planctomycetaceae;unclassified |
| 95 | Bacteria;Planctomycetes;Planctomycetacia;Planctomycetales;Planctomycetaceae;unclassified |
| 96 | Bacteria;Planctomycetes;Planctomycetacia;Planctomycetales;Planctomycetaceae;Blastopirellula |
| 97 | Bacteria;Planctomycetes;OM190;unclassified;unclassified;unclassified |
| 98 | Bacteria;Planctomycetes;OM190;unclassified;unclassified;unclassified |
| 99 | Bacteria;Planctomycetes;Phycisphaerae;Phycisphaerales;Phycisphaeraceae;SM1A02 |
| 100 | Bacteria;Hydrogenedentes;unclassified;unclassified;unclassified;unclassified |
| 101 | Bacteria;Proteobacteria;Gammaproteobacteria;Alteromonadales;Shewanellaceae;Shewanella |
| 102 | Bacteria;Proteobacteria;Gammaproteobacteria;Aeromonadales;Aeromonadaceae;unclassified |
| 103 | Bacteria;Proteobacteria;Gammaproteobacteria;Vibrionales;Vibrionaceae;Vibrio |
| 104 | Bacteria;Proteobacteria;Gammaproteobacteria;Alteromonadales;Alteromonadaceae;Alteromonas |
| 105 | Bacteria;Proteobacteria;Gammaproteobacteria;Alteromonadales;Alteromonadaceae;Paraglaciecola |
| 106 | Bacteria;Proteobacteria;Gammaproteobacteria;Cellvibrionales;Cellvibrionaceae;Cellvibrio |
| 107 | Bacteria;Proteobacteria;Gammaproteobacteria;Cellvibrionales;Cellvibrionaceae;Cellvibrio |
| 108 | Bacteria;Proteobacteria;Gammaproteobacteria;Xanthomonadales;Xanthomonadaceae;Aquimonas |
| 109 | Bacteria;Proteobacteria;Gammaproteobacteria;Xanthomonadales;Xanthomonadaceae;unclassified |
| 110 | Bacteria;Proteobacteria;Gammaproteobacteria;Cellvibrionales;Halieaceae;unclassified |
| 111 | Bacteria;Proteobacteria;Gammaproteobacteria;Cellvibrionales;Halieaceae;OM60(NOR5)_clade |
| 112 | Bacteria;Proteobacteria;Gammaproteobacteria;Pseudomonadales;Moraxellaceae;unclassified |
| 113 | Bacteria;Proteobacteria;Gammaproteobacteria;Pseudomonadales;Moraxellaceae;Acinetobacter |
| 114 | Bacteria;Proteobacteria;Gammaproteobacteria;Pseudomonadales;Pseudomonadaceae;Pseudomonas |
| 115 | Bacteria;Proteobacteria;Gammaproteobacteria;Pseudomonadales;Pseudomonadaceae;Pseudomonas |
| 116 | Bacteria;Proteobacteria;Gammaproteobacteria;Oceanospirillales;SAR86_clade;unclassified |
| 117 | Bacteria;Proteobacteria;Gammaproteobacteria;Order_Incertae_Sedis;Family_Incertae_Sedis;Marinicella |
| 118 | Bacteria;Proteobacteria;Gammaproteobacteria;Cellvibrionales;Cellvibrionaceae;unclassified |
| 119 | Bacteria;Proteobacteria;Gammaproteobacteria;Cellvibrionales;Cellvibrionaceae;unclassified |
| 120 | Bacteria;Proteobacteria;Gammaproteobacteria;Xanthomonadales;JTB255_marine_benthic_group;unclassified |
| 121 | Bacteria;Proteobacteria;Gammaproteobacteria;Oceanospirillales;Oceanospirillaceae;Neptunomonas |
| 122 | Bacteria;Proteobacteria;Betaproteobacteria;Burkholderiales;Comamonadaceae;Hydrogenophaga |
| 123 | Bacteria;Proteobacteria;Betaproteobacteria;Burkholderiales;Comamonadaceae;Hydrogenophaga |
| 124 | Bacteria;Proteobacteria;Betaproteobacteria;Burkholderiales;Comamonadaceae;unclassified |
| 125 | Bacteria;Proteobacteria;Betaproteobacteria;Burkholderiales;Comamonadaceae;unclassified |
| 126 | Bacteria;Proteobacteria;Betaproteobacteria;Burkholderiales;Comamonadaceae;unclassified |
| 127 | Bacteria;Proteobacteria;Betaproteobacteria;Burkholderiales;Comamonadaceae;unclassified |
| 128 | Bacteria;Proteobacteria;Betaproteobacteria;Burkholderiales;Comamonadaceae;unclassified |
| 129 | Bacteria;Proteobacteria;Betaproteobacteria;Burkholderiales;Comamonadaceae;unclassified |
| 130 | Bacteria;Proteobacteria;Betaproteobacteria;Burkholderiales;Comamonadaceae;BAL58_marine_group |
| 131 | Bacteria;Proteobacteria;Betaproteobacteria;Rhodocyclales;Rhodocyclaceae;Zoogloea |
| 132 | Bacteria;Proteobacteria;Betaproteobacteria;Burkholderiales;Burkholderiaceae;Limnobacter |
| 133 | Bacteria;Proteobacteria;Betaproteobacteria;Burkholderiales;unclassified;unclassified |
| 134 | Bacteria;Proteobacteria;Betaproteobacteria;Rhodocyclales;Rhodocyclaceae;Sulfuritalea |
| 135 | Bacteria;Proteobacteria;Betaproteobacteria;Rhodocyclales;Rhodocyclaceae;Candidatus_Accumulibacter |
| 136 | Bacteria;Proteobacteria;Betaproteobacteria;Rhodocyclales;Rhodocyclaceae;Candidatus_Accumulibacter |
| 137 | Bacteria;Proteobacteria;Betaproteobacteria;Methylophilales;Methylophilaceae;Methylotenera |
| 138 | Bacteria;Proteobacteria;Betaproteobacteria;Methylophilales;Methylophilaceae;Methylophilus |
| 139 | Bacteria;Proteobacteria;Betaproteobacteria;unclassified;unclassified;unclassified |
| 140 | Bacteria;Proteobacteria;Betaproteobacteria;Burkholderiales;Alcaligenaceae;unclassified |
| 141 | Bacteria;Proteobacteria;Betaproteobacteria;unclassified;unclassified;unclassified |
| 142 | Bacteria;Proteobacteria;Deltaproteobacteria;Oligoflexales;Oligoflexaceae;unclassified |
| 143 | Bacteria;Proteobacteria;Gammaproteobacteria;Run-SP154;unclassified;unclassified |
| 144 | Bacteria;Proteobacteria;Epsilonproteobacteria;Campylobacterales;Campylobacteraceae;Arcobacter |
| 145 | Bacteria;Proteobacteria;Epsilonproteobacteria;Campylobacterales;Campylobacteraceae;Arcobacter |
| 146 | Bacteria;Proteobacteria;Epsilonproteobacteria;Campylobacterales;Campylobacteraceae;Arcobacter |
| 147 | Bacteria;Proteobacteria;Alphaproteobacteria;Rickettsiales;Rickettsiaceae;unclassified |
| 148 | Bacteria;Proteobacteria;Alphaproteobacteria;Sphingomonadales;Sphingomonadaceae;unclassified |
| 149 | Bacteria;Proteobacteria;Alphaproteobacteria;Sphingomonadales;Sphingomonadaceae;Sphingopyxis |
| 150 | Bacteria;Proteobacteria;Alphaproteobacteria;Sphingomonadales;Erythrobacteraceae;unclassified |
| 151 | Bacteria;Proteobacteria;Alphaproteobacteria;Sphingomonadales;unclassified;unclassified |
| 152 | Bacteria;Proteobacteria;Alphaproteobacteria;Sphingomonadales;Erythrobacteraceae;Erythrobacter |
| 153 | Bacteria;Proteobacteria;Alphaproteobacteria;Sphingomonadales;Erythrobacteraceae;Erythrobacter |
| 154 | Bacteria;Proteobacteria;Alphaproteobacteria;Caulobacterales;Caulobacteraceae;Brevundimonas |
| 155 | Bacteria;Proteobacteria;Alphaproteobacteria;Caulobacterales;Hyphomonadaceae;Hyphomonas |
| 156 | Bacteria;Proteobacteria;Alphaproteobacteria;Caulobacterales;Hyphomonadaceae;Hyphomonas |
| 157 | Bacteria;Proteobacteria;Alphaproteobacteria;Caulobacterales;Hyphomonadaceae;Hirschia |
| 158 | Bacteria;Proteobacteria;Alphaproteobacteria;Caulobacterales;Hyphomonadaceae;unclassified |
| 159 | Bacteria;Proteobacteria;Alphaproteobacteria;Rhodobacterales;Rhodobacteraceae;Defluviimonas |
| 160 | Bacteria;Proteobacteria;Alphaproteobacteria;Rhodobacterales;Rhodobacteraceae;unclassified |
| 161 | Bacteria;Proteobacteria;Alphaproteobacteria;Rhodobacterales;Rhodobacteraceae;unclassified |
| 162 | Bacteria;Proteobacteria;Alphaproteobacteria;Rhodobacterales;Rhodobacteraceae;unclassified |
| 163 | Bacteria;Proteobacteria;Alphaproteobacteria;Rhodobacterales;Rhodobacteraceae;unclassified |
| 164 | Bacteria;Proteobacteria;Alphaproteobacteria;Rhodobacterales;Rhodobacteraceae;unclassified |
| 165 | Bacteria;Proteobacteria;Alphaproteobacteria;Rhodobacterales;Rhodobacteraceae;unclassified |
| 166 | Bacteria;Proteobacteria;Alphaproteobacteria;Rhodobacterales;Rhodobacteraceae;unclassified |
| 167 | Bacteria;Proteobacteria;Alphaproteobacteria;Rhodobacterales;Rhodobacteraceae;unclassified |
| 168 | Bacteria;Proteobacteria;Alphaproteobacteria;Rhodobacterales;Rhodobacteraceae;unclassified |
| 169 | Bacteria;Proteobacteria;Alphaproteobacteria;Rhodobacterales;Rhodobacteraceae;unclassified |
| 170 | Bacteria;Proteobacteria;Alphaproteobacteria;Rhodobacterales;Rhodobacteraceae;unclassified |
| 171 | Bacteria;Proteobacteria;Alphaproteobacteria;Rhodobacterales;Rhodobacteraceae;unclassified |
| 172 | Bacteria;Proteobacteria;Alphaproteobacteria;Rhizobiales;Phyllobacteriaceae;unclassified |
| 173 | Bacteria;Proteobacteria;Alphaproteobacteria;Rhizobiales;Phyllobacteriaceae;Hoeflea |
| 174 | Bacteria;Proteobacteria;Alphaproteobacteria;Rhodobacterales;Rhodobacteraceae;Marivita |
| 175 | Bacteria;Proteobacteria;Alphaproteobacteria;Rhodobacterales;Rhodobacteraceae;unclassified |

**Additional file 5**: Linear discriminant analyses (using LEfSe) identified differentially abundant bacterial OTUs in plastic-associated assemblages compared to wood-associated and water communities (free-living and attached). Analyses are based on OTUs (16S rRNA gene data) with an average relative abundance of ≥0.1%. Listed are significantly differentially abundant taxa from the individual datasets ‘Baltic’, ‘Warnow’ and ‘WWTP’ and corresponding logarithmized LDA values. uc:unclassified.

| **Differentially abundant feature** | **log LDA** |
| --- | --- |
| **Dataset Baltic** |  |
| Bacteria.Bacteroidetes | 5.072 |
| Bacteria.Bacteroidetes.Cytophagia | 3.109 |
| Bacteria.Bacteroidetes.Cytophagia.Cytophagales | 3.109 |
| Bacteria.Bacteroidetes.Cytophagia.Cytophagales.Cyclobacteriaceae | 3.052 |
| Bacteria.Bacteroidetes.Cytophagia.Cytophagales.Cyclobacteriaceae.Algoriphagus | 3.052 |
| Bacteria.Bacteroidetes.Cytophagia.Cytophagales.Cyclobacteriaceae.Algoriphagus.Otu000127 | 3.052 |
| Bacteria.Bacteroidetes.Flavobacteriia | 5.028 |
| Bacteria.Bacteroidetes.Flavobacteriia.Flavobacteriales | 5.049 |
| Bacteria.Bacteroidetes.Flavobacteriia.Flavobacteriales.Cryomorphaceae.Fluviicola | 3.534 |
| Bacteria.Bacteroidetes.Flavobacteriia.Flavobacteriales.Cryomorphaceae.Fluviicola.Otu000066 | 3.534 |
| Bacteria.Bacteroidetes.Flavobacteriia.Flavobacteriales.Flavobacteriaceae | 5.001 |
| Bacteria.Bacteroidetes.Flavobacteriia.Flavobacteriales.Flavobacteriaceae.Aquibacter.Otu000062 | 3.921 |
| Bacteria.Bacteroidetes.Flavobacteriia.Flavobacteriales.Flavobacteriaceae.Dokdonia | 3.974 |
| Bacteria.Bacteroidetes.Flavobacteriia.Flavobacteriales.Flavobacteriaceae.Dokdonia.Otu000063 | 3.974 |
| Bacteria.Bacteroidetes.Flavobacteriia.Flavobacteriales.Flavobacteriaceae.Gaetbulibacter | 3.097 |
| Bacteria.Bacteroidetes.Flavobacteriia.Flavobacteriales.Flavobacteriaceae.Gaetbulibacter.Otu000086 | 3.097 |
| Bacteria.Bacteroidetes.Flavobacteriia.Flavobacteriales.Flavobacteriaceae.Tenacibaculum.Otu000083 | 3.302 |
| Bacteria.Bacteroidetes.Flavobacteriia.Flavobacteriales.Flavobacteriaceae.uc | 5.157 |
| Bacteria.Bacteroidetes.Flavobacteriia.Flavobacteriales.Flavobacteriaceae.uc.Otu000001 | 4.894 |
| Bacteria.Bacteroidetes.Flavobacteriia.Flavobacteriales.Flavobacteriaceae.uc.Otu000003 | 4.722 |
| Bacteria.Bacteroidetes.Flavobacteriia.Flavobacteriales.Flavobacteriaceae.uc.Otu000020 | 4.103 |
| Bacteria.Bacteroidetes.Flavobacteriia.Flavobacteriales.Flavobacteriaceae.uc.Otu000033 | 3.465 |
| Bacteria.Bacteroidetes.Flavobacteriia.Flavobacteriales.Flavobacteriaceae.uc.Otu000093 | 3.487 |
| Bacteria.Bacteroidetes.Flavobacteriia.Flavobacteriales.Schleiferiaceae | 3.228 |
| Bacteria.Bacteroidetes.Flavobacteriia.Flavobacteriales.Schleiferiaceae.Schleiferia | 3.228 |
| Bacteria.Bacteroidetes.Flavobacteriia.Flavobacteriales.Schleiferiaceae.Schleiferia.Otu000077 | 3.228 |
| Bacteria.Bacteroidetes.Flavobacteriia.Flavobacteriales.uc | 4.163 |
| Bacteria.Bacteroidetes.Flavobacteriia.Flavobacteriales.uc.uc | 4.163 |
| Bacteria.Bacteroidetes.Flavobacteriia.Flavobacteriales.uc.uc.Otu000040 | 3.886 |
| Bacteria.Bacteroidetes.Flavobacteriia.Flavobacteriales.uc.uc.Otu000078 | 3.717 |
| Bacteria.Bacteroidetes.Flavobacteriia.Flavobacteriales.uc.uc.Otu000126 | 3.241 |
| Bacteria.Bacteroidetes.Sphingobacteriia | 4.129 |
| Bacteria.Bacteroidetes.Sphingobacteriia.Sphingobacteriales | 4.129 |
| Bacteria.Bacteroidetes.Sphingobacteriia.Sphingobacteriales.Saprospiraceae | 4.150 |
| Bacteria.Bacteroidetes.Sphingobacteriia.Sphingobacteriales.Saprospiraceae.uc | 4.077 |
| Bacteria.Bacteroidetes.Sphingobacteriia.Sphingobacteriales.Saprospiraceae.uc.Otu000037 | 3.501 |
| Bacteria.Bacteroidetes.Sphingobacteriia.Sphingobacteriales.Saprospiraceae.uc.Otu000070 | 3.906 |
| Bacteria.Bacteroidetes.Sphingobacteriia.Sphingobacteriales.Saprospiraceae.uc.Otu000088 | 2.940 |
| Bacteria.Bacteroidetes.uc | 3.695 |
| Bacteria.Bacteroidetes.uc.uc | 3.695 |
| Bacteria.Bacteroidetes.uc.uc.uc | 3.695 |
| Bacteria.Bacteroidetes.uc.uc.uc.uc | 3.695 |
| Bacteria.Bacteroidetes.uc.uc.uc.uc.Otu000065 | 3.657 |
| Bacteria.Planctomycetes.OM190 | 4.148 |
| Bacteria.Planctomycetes.OM190.uc | 4.148 |
| Bacteria.Planctomycetes.OM190.uc.uc | 4.148 |
| Bacteria.Planctomycetes.OM190.uc.uc.uc | 4.148 |
| Bacteria.Planctomycetes.OM190.uc.uc.uc.Otu000028 | 4.100 |
| Bacteria.Planctomycetes.OM190.uc.uc.uc.Otu000079 | 3.173 |
| Bacteria.Planctomycetes.Phycisphaerae | 3.582 |
| Bacteria.Planctomycetes.Phycisphaerae.Phycisphaerales | 3.582 |
| Bacteria.Planctomycetes.Phycisphaerae.Phycisphaerales.Phycisphaeraceae | 3.582 |
| Bacteria.Planctomycetes.Phycisphaerae.Phycisphaerales.Phycisphaeraceae.SM1A02 | 3.582 |
| Bacteria.Planctomycetes.Phycisphaerae.Phycisphaerales.Phycisphaeraceae.SM1A02.Otu000050 | 3.582 |
| Bacteria.Planctomycetes.Planctomycetacia.Planctomycetales.Planctomycetaceae.Blastopirellula | 4.288 |
| Bacteria.Planctomycetes.Planctomycetacia.Planctomycetales.Planctomycetaceae.Blastopirellula.Otu000017 | 4.288 |
| Bacteria.Planctomycetes.Planctomycetacia.Planctomycetales.Planctomycetaceae.Pirellula | 3.253 |
| Bacteria.Planctomycetes.Planctomycetacia.Planctomycetales.Planctomycetaceae.Pirellula.Otu000091 | 3.251 |
| Bacteria.Planctomycetes.Planctomycetacia.Planctomycetales.Planctomycetaceae.Planctomyces.Otu000076 | 3.795 |
| Bacteria.Planctomycetes.Planctomycetacia.Planctomycetales.Planctomycetaceae.Planctomyces.Otu000098 | 3.236 |
| Bacteria.Planctomycetes.Planctomycetacia.Planctomycetales.Planctomycetaceae.Planctomyces.Otu000100 | 3.447 |
| Bacteria.Planctomycetes.Planctomycetacia.Planctomycetales.Planctomycetaceae.uc | 4.062 |
| Bacteria.Planctomycetes.Planctomycetacia.Planctomycetales.Planctomycetaceae.uc.Otu000039 | 3.602 |
| Bacteria.Planctomycetes.Planctomycetacia.Planctomycetales.Planctomycetaceae.uc.Otu000060 | 3.398 |
| Bacteria.Proteobacteria.Alphaproteobacteria.Caulobacterales | 4.424 |
| Bacteria.Proteobacteria.Alphaproteobacteria.Caulobacterales.Hyphomonadaceae | 4.431 |
| Bacteria.Proteobacteria.Alphaproteobacteria.Caulobacterales.Hyphomonadaceae.Hyphomonas | 4.207 |
| Bacteria.Proteobacteria.Alphaproteobacteria.Caulobacterales.Hyphomonadaceae.Hyphomonas.Otu000022 | 4.085 |
| Bacteria.Proteobacteria.Alphaproteobacteria.Caulobacterales.Hyphomonadaceae.Hyphomonas.Otu000043 | 3.606 |
| Bacteria.Proteobacteria.Alphaproteobacteria.Caulobacterales.Hyphomonadaceae.uc | 4.035 |
| Bacteria.Proteobacteria.Alphaproteobacteria.Caulobacterales.Hyphomonadaceae.uc.Otu000029 | 4.035 |
| Bacteria.Proteobacteria.Alphaproteobacteria.Rhizobiales | 3.991 |
| Bacteria.Proteobacteria.Alphaproteobacteria.Rhizobiales.Phyllobacteriaceae | 3.991 |
| Bacteria.Proteobacteria.Alphaproteobacteria.Rhizobiales.Phyllobacteriaceae.uc | 3.991 |
| Bacteria.Proteobacteria.Alphaproteobacteria.Rhizobiales.Phyllobacteriaceae.uc.Otu000047 | 3.912 |
| Bacteria.Proteobacteria.Alphaproteobacteria.Rhizobiales.Phyllobacteriaceae.uc.Otu000057 | 3.227 |
| Bacteria.Proteobacteria.Alphaproteobacteria.Rhodobacterales.Rhodobacteraceae.uc | 4.610 |
| Bacteria.Proteobacteria.Alphaproteobacteria.Rhodobacterales.Rhodobacteraceae.uc.Otu000005 | 4.090 |
| Bacteria.Proteobacteria.Alphaproteobacteria.Rhodobacterales.Rhodobacteraceae.uc.Otu000006 | 4.159 |
| Bacteria.Proteobacteria.Alphaproteobacteria.Rhodobacterales.Rhodobacteraceae.uc.Otu000021 | 3.666 |
| Bacteria.Proteobacteria.Alphaproteobacteria.Rhodobacterales.Rhodobacteraceae.uc.Otu000069 | 3.522 |
| Bacteria.Proteobacteria.Alphaproteobacteria.Rhodobacterales.Rhodobacteraceae.uc.Otu000115 | 3.008 |
| Bacteria.Proteobacteria.Alphaproteobacteria.Rhodobacterales.Rhodobacteraceae.uc.Otu000125 | 3.024 |
| Bacteria.Proteobacteria.Alphaproteobacteria.Rhodobacterales.Rhodobacteraceae.uc.Otu000128 | 3.443 |
| Bacteria.Proteobacteria.Alphaproteobacteria.Sphingomonadales | 4.096 |
| Bacteria.Proteobacteria.Alphaproteobacteria.Sphingomonadales.Erythrobacteraceae | 4.096 |
| Bacteria.Proteobacteria.Alphaproteobacteria.Sphingomonadales.Erythrobacteraceae.Erythrobacter | 3.896 |
| Bacteria.Proteobacteria.Alphaproteobacteria.Sphingomonadales.Erythrobacteraceae.Erythrobacter.Otu000030 | 3.731 |
| Bacteria.Proteobacteria.Alphaproteobacteria.Sphingomonadales.Erythrobacteraceae.Erythrobacter.Otu000117 | 3.421 |
| Bacteria.Proteobacteria.Alphaproteobacteria.Sphingomonadales.Erythrobacteraceae.uc | 3.674 |
| Bacteria.Proteobacteria.Alphaproteobacteria.Sphingomonadales.Erythrobacteraceae.uc.Otu000089 | 3.674 |
| Bacteria.Proteobacteria.Betaproteobacteria | 4.536 |
| Bacteria.Proteobacteria.Betaproteobacteria.Burkholderiales.uc | 3.530 |
| Bacteria.Proteobacteria.Betaproteobacteria.Burkholderiales.uc.uc | 3.530 |
| Bacteria.Proteobacteria.Betaproteobacteria.Burkholderiales.uc.uc.Otu000068 | 3.530 |
| Bacteria.Proteobacteria.Betaproteobacteria.Methylophilales | 4.501 |
| Bacteria.Proteobacteria.Betaproteobacteria.Methylophilales.Methylophilaceae | 4.501 |
| Bacteria.Proteobacteria.Betaproteobacteria.Methylophilales.Methylophilaceae.Methylotenera | 4.501 |
| Bacteria.Proteobacteria.Betaproteobacteria.Methylophilales.Methylophilaceae.Methylotenera.Otu000004 | 4.501 |
| Bacteria.Proteobacteria.Gammaproteobacteria.Order_Incertae_Sedis | 3.636 |
| Bacteria.Proteobacteria.Gammaproteobacteria.Order_Incertae_Sedis.Family_Incertae_Sedis | 3.636 |
| Bacteria.Proteobacteria.Gammaproteobacteria.Order_Incertae_Sedis.Family_Incertae_Sedis.Marinicella | 3.636 |
| Bacteria.Proteobacteria.Gammaproteobacteria.Order_Incertae_Sedis.Family_Incertae_Sedis.Marinicella.Otu000059 | 3.636 |
| Bacteria.Verrucomicrobia.Verrucomicrobiae.Verrucomicrobiales.DEV007 | 3.660 |
| Bacteria.Verrucomicrobia.Verrucomicrobiae.Verrucomicrobiales.DEV007.uc | 3.660 |
| Bacteria.Verrucomicrobia.Verrucomicrobiae.Verrucomicrobiales.DEV007.uc.Otu000073 | 3.410 |
| **Dataset Warnow** |  |
| Bacteria.Bacteroidetes.Cytophagia.Cytophagales.Cytophagaceae.Leadbetterella.Otu000051 | 3.993 |
| Bacteria.Bacteroidetes.Flavobacteriia.Flavobacteriales.Cryomorphaceae.Fluviicola | 3.672 |
| Bacteria.Bacteroidetes.Flavobacteriia.Flavobacteriales.Cryomorphaceae.Fluviicola.Otu000066 | 3.672 |
| Bacteria.Bacteroidetes.Flavobacteriia.Flavobacteriales.Flavobacteriaceae.Aquibacter.Otu000062 | 3.287 |
| Bacteria.Bacteroidetes.Flavobacteriia.Flavobacteriales.Flavobacteriaceae.Cellulophaga | 3.887 |
| Bacteria.Bacteroidetes.Flavobacteriia.Flavobacteriales.Flavobacteriaceae.Cellulophaga.Otu000034 | 3.887 |
| Bacteria.Bacteroidetes.Flavobacteriia.Flavobacteriales.Flavobacteriaceae.Flavobacterium.Otu000013 | 4.409 |
| Bacteria.Bacteroidetes.Flavobacteriia.Flavobacteriales.Flavobacteriaceae.Tenacibaculum.Otu000083 | 3.296 |
| Bacteria.Bacteroidetes.Flavobacteriia.Flavobacteriales.Flavobacteriaceae.uc | 4.858 |
| Bacteria.Bacteroidetes.Flavobacteriia.Flavobacteriales.Flavobacteriaceae.uc.Otu000003 | 4.401 |
| Bacteria.Bacteroidetes.Flavobacteriia.Flavobacteriales.Flavobacteriaceae.uc.Otu000020 | 3.790 |
| Bacteria.Bacteroidetes.Flavobacteriia.Flavobacteriales.Flavobacteriaceae.uc.Otu000033 | 4.005 |
| Bacteria.Bacteroidetes.Flavobacteriia.Flavobacteriales.Flavobacteriaceae.uc.Otu000093 | 3.561 |
| Bacteria.Bacteroidetes.Flavobacteriia.Flavobacteriales.Flavobacteriaceae.uc.Otu000113 | 3.405 |
| Bacteria.Bacteroidetes.Flavobacteriia.Flavobacteriales.uc.uc.Otu000040 | 3.387 |
| Bacteria.Bacteroidetes.Sphingobacteriia.Sphingobacteriales.Saprospiraceae.uc.Otu000037 | 3.309 |
| Bacteria.Bacteroidetes.Sphingobacteriia.Sphingobacteriales.Saprospiraceae.uc.Otu000088 | 3.986 |
| Bacteria.Bacteroidetes.uc.uc.uc.uc.Otu000065 | 3.153 |
| Bacteria.Planctomycetes.OM190.uc.uc.uc.Otu000028 | 3.454 |
| Bacteria.Planctomycetes.Phycisphaerae | 3.076 |
| Bacteria.Planctomycetes.Phycisphaerae.Phycisphaerales | 3.076 |
| Bacteria.Planctomycetes.Phycisphaerae.Phycisphaerales.Phycisphaeraceae | 3.076 |
| Bacteria.Planctomycetes.Phycisphaerae.Phycisphaerales.Phycisphaeraceae.SM1A02 | 3.076 |
| Bacteria.Planctomycetes.Phycisphaerae.Phycisphaerales.Phycisphaeraceae.SM1A02.Otu000050 | 3.076 |
| Bacteria.Planctomycetes.Planctomycetacia.Planctomycetales.Planctomycetaceae.Blastopirellula | 3.518 |
| Bacteria.Planctomycetes.Planctomycetacia.Planctomycetales.Planctomycetaceae.Blastopirellula.Otu000017 | 3.518 |
| Bacteria.Planctomycetes.Planctomycetacia.Planctomycetales.Planctomycetaceae.Pirellula | 3.612 |
| Bacteria.Planctomycetes.Planctomycetacia.Planctomycetales.Planctomycetaceae.Pirellula.Otu000091 | 3.606 |
| Bacteria.Planctomycetes.Planctomycetacia.Planctomycetales.Planctomycetaceae.Planctomyces.Otu000076 | 3.409 |
| Bacteria.Planctomycetes.Planctomycetacia.Planctomycetales.Planctomycetaceae.Planctomyces.Otu000100 | 3.337 |
| Bacteria.Planctomycetes.Planctomycetacia.Planctomycetales.Planctomycetaceae.uc | 3.617 |
| Bacteria.Proteobacteria.Alphaproteobacteria | 5.021 |
| Bacteria.Proteobacteria.Alphaproteobacteria.Caulobacterales | 4.144 |
| Bacteria.Proteobacteria.Alphaproteobacteria.Caulobacterales.Caulobacteraceae | 3.185 |
| Bacteria.Proteobacteria.Alphaproteobacteria.Caulobacterales.Caulobacteraceae.Brevundimonas | 3.187 |
| Bacteria.Proteobacteria.Alphaproteobacteria.Caulobacterales.Caulobacteraceae.Brevundimonas.Otu000216 | 3.185 |
| Bacteria.Proteobacteria.Alphaproteobacteria.Caulobacterales.Hyphomonadaceae | 4.112 |
| Bacteria.Proteobacteria.Alphaproteobacteria.Caulobacterales.Hyphomonadaceae.Hyphomonas.Otu000043 | 3.665 |
| Bacteria.Proteobacteria.Alphaproteobacteria.Caulobacterales.Hyphomonadaceae.uc | 3.741 |
| Bacteria.Proteobacteria.Alphaproteobacteria.Caulobacterales.Hyphomonadaceae.uc.Otu000029 | 3.741 |
| Bacteria.Proteobacteria.Alphaproteobacteria.Rhizobiales | 3.991 |
| Bacteria.Proteobacteria.Alphaproteobacteria.Rhizobiales.Phyllobacteriaceae | 3.990 |
| Bacteria.Proteobacteria.Alphaproteobacteria.Rhizobiales.Phyllobacteriaceae.uc | 3.989 |
| Bacteria.Proteobacteria.Alphaproteobacteria.Rhizobiales.Phyllobacteriaceae.uc.Otu000047 | 3.461 |
| Bacteria.Proteobacteria.Alphaproteobacteria.Rhizobiales.Phyllobacteriaceae.uc.Otu000057 | 3.934 |
| Bacteria.Proteobacteria.Alphaproteobacteria.Rhodobacterales | 4.896 |
| Bacteria.Proteobacteria.Alphaproteobacteria.Rhodobacterales.Rhodobacteraceae | 4.896 |
| Bacteria.Proteobacteria.Alphaproteobacteria.Rhodobacterales.Rhodobacteraceae.uc | 4.996 |
| Bacteria.Proteobacteria.Alphaproteobacteria.Rhodobacterales.Rhodobacteraceae.uc.Otu000006 | 4.638 |
| Bacteria.Proteobacteria.Alphaproteobacteria.Rhodobacterales.Rhodobacteraceae.uc.Otu000021 | 4.093 |
| Bacteria.Proteobacteria.Alphaproteobacteria.Rhodobacterales.Rhodobacteraceae.uc.Otu000027 | 4.066 |
| Bacteria.Proteobacteria.Alphaproteobacteria.Rhodobacterales.Rhodobacteraceae.uc.Otu000069 | 3.584 |
| Bacteria.Proteobacteria.Alphaproteobacteria.Rhodobacterales.Rhodobacteraceae.uc.Otu000128 | 3.436 |
| Bacteria.Proteobacteria.Alphaproteobacteria.Rhodobacterales.Rhodobacteraceae.uc.Otu000148 | 3.279 |
| Bacteria.Proteobacteria.Alphaproteobacteria.Sphingomonadales | 4.029 |
| Bacteria.Proteobacteria.Alphaproteobacteria.Sphingomonadales.Erythrobacteraceae | 3.509 |
| Bacteria.Proteobacteria.Alphaproteobacteria.Sphingomonadales.Erythrobacteraceae.Erythrobacter | 3.442 |
| Bacteria.Proteobacteria.Alphaproteobacteria.Sphingomonadales.Erythrobacteraceae.Erythrobacter.Otu000030 | 3.463 |
| Bacteria.Proteobacteria.Alphaproteobacteria.Sphingomonadales.uc | 3.597 |
| Bacteria.Proteobacteria.Alphaproteobacteria.Sphingomonadales.uc.uc | 3.597 |
| Bacteria.Proteobacteria.Alphaproteobacteria.Sphingomonadales.uc.uc.Otu000173 | 3.598 |
| Bacteria.Proteobacteria.Betaproteobacteria.Burkholderiales.Comamonadaceae.Hydrogenophaga.Otu000009 | 4.426 |
| Bacteria.Proteobacteria.Betaproteobacteria.Burkholderiales.uc | 3.438 |
| Bacteria.Proteobacteria.Betaproteobacteria.Burkholderiales.uc.uc | 3.441 |
| Bacteria.Proteobacteria.Betaproteobacteria.Burkholderiales.uc.uc.Otu000068 | 3.442 |
| Bacteria.Proteobacteria.Gammaproteobacteria.Cellvibrionales.Halieaceae | 3.796 |
| Bacteria.Proteobacteria.Gammaproteobacteria.Cellvibrionales.Halieaceae.Halioglobus | 3.648 |
| Bacteria.Proteobacteria.Gammaproteobacteria.Cellvibrionales.Halieaceae.Halioglobus.Otu000053 | 3.646 |
| Bacteria.Proteobacteria.Gammaproteobacteria.Order_Incertae_Sedis | 3.628 |
| Bacteria.Proteobacteria.Gammaproteobacteria.Order_Incertae_Sedis.Family_Incertae_Sedis | 3.628 |
| Bacteria.Proteobacteria.Gammaproteobacteria.Order_Incertae_Sedis.Family_Incertae_Sedis.Marinicella | 3.628 |
| Bacteria.Proteobacteria.Gammaproteobacteria.Order_Incertae_Sedis.Family_Incertae_Sedis.Marinicella.Otu000059 | 3.628 |
| Bacteria.Proteobacteria.Gammaproteobacteria.Xanthomonadales | 3.059 |
| Bacteria.Proteobacteria.Gammaproteobacteria.Xanthomonadales.JTB255_marine_benthic_group | 3.052 |
| Bacteria.Proteobacteria.Gammaproteobacteria.Xanthomonadales.JTB255_marine_benthic_group.uc | 3.051 |
| Bacteria.Proteobacteria.Gammaproteobacteria.Xanthomonadales.JTB255_marine_benthic_group.uc.Otu000135 | 3.052 |
| Bacteria.Verrucomicrobia.Verrucomicrobiae.Verrucomicrobiales.DEV007 | 3.363 |
| Bacteria.Verrucomicrobia.Verrucomicrobiae.Verrucomicrobiales.DEV007.uc | 3.363 |
| Bacteria.Verrucomicrobia.Verrucomicrobiae.Verrucomicrobiales.DEV007.uc.Otu000061 | 3.222 |
| Bacteria.Verrucomicrobia.Verrucomicrobiae.Verrucomicrobiales.DEV007.uc.Otu000073 | 3.512 |
| **Dataset WWTP** |  |
| Bacteria.Bacteroidetes | 5.158 |
| Bacteria.Bacteroidetes.Flavobacteriia | 5.027 |
| Bacteria.Bacteroidetes.Flavobacteriia.Flavobacteriales | 5.027 |
| Bacteria.Bacteroidetes.Flavobacteriia.Flavobacteriales.Flavobacteriaceae | 5.025 |
| Bacteria.Bacteroidetes.Flavobacteriia.Flavobacteriales.Flavobacteriaceae.Flavobacterium | 5.016 |
| Bacteria.Bacteroidetes.Flavobacteriia.Flavobacteriales.Flavobacteriaceae.Flavobacterium.Otu000011 | 3.190 |
| Bacteria.Bacteroidetes.Flavobacteriia.Flavobacteriales.Flavobacteriaceae.Flavobacterium.Otu000038 | 4.154 |
| Bacteria.Bacteroidetes.Flavobacteriia.Flavobacteriales.Flavobacteriaceae.Flavobacterium.Otu000046 | 4.247 |
| Bacteria.Bacteroidetes.Flavobacteriia.Flavobacteriales.Flavobacteriaceae.Flavobacterium.Otu000056 | 3.953 |
| Bacteria.Bacteroidetes.Flavobacteriia.Flavobacteriales.Flavobacteriaceae.Flavobacterium.Otu000130 | 4.221 |
| Bacteria.Bacteroidetes.Flavobacteriia.Flavobacteriales.Flavobacteriaceae.Flavobacterium.Otu000289 | 3.872 |
| Bacteria.Bacteroidetes.Flavobacteriia.Flavobacteriales.Flavobacteriaceae.Tenacibaculum | 3.224 |
| Bacteria.Bacteroidetes.Flavobacteriia.Flavobacteriales.Flavobacteriaceae.Tenacibaculum.Otu000072 | 3.217 |
| Bacteria.Bacteroidetes.Flavobacteriia.Flavobacteriales.Flavobacteriaceae.uc | 3.309 |
| Bacteria.Bacteroidetes.Flavobacteriia.Flavobacteriales.Flavobacteriaceae.uc.Otu000003 | 3.270 |
| Bacteria.Bacteroidetes.Sphingobacteriia.Sphingobacteriales.Chitinophagaceae | 3.902 |
| Bacteria.Bacteroidetes.Sphingobacteriia.Sphingobacteriales.Chitinophagaceae.Terrimonas | 3.902 |
| Bacteria.Bacteroidetes.Sphingobacteriia.Sphingobacteriales.Chitinophagaceae.Terrimonas.Otu000169 | 3.902 |
| Bacteria.Bacteroidetes.Sphingobacteriia.Sphingobacteriales.Saprospiraceae.uc.Otu000167 | 3.817 |
| Bacteria.Planctomycetes | 4.123 |
| Bacteria.Planctomycetes.Planctomycetacia | 4.127 |
| Bacteria.Planctomycetes.Planctomycetacia.Planctomycetales | 4.127 |
| Bacteria.Planctomycetes.Planctomycetacia.Planctomycetales.Planctomycetaceae | 4.127 |
| Bacteria.Planctomycetes.Planctomycetacia.Planctomycetales.Planctomycetaceae.Planctomyces | 3.897 |
| Bacteria.Planctomycetes.Planctomycetacia.Planctomycetales.Planctomycetaceae.Planctomyces.Otu000076 | 3.446 |
| Bacteria.Planctomycetes.Planctomycetacia.Planctomycetales.Planctomycetaceae.Planctomyces.Otu000186 | 3.816 |
| Bacteria.Proteobacteria.Alphaproteobacteria | 5.058 |
| Bacteria.Proteobacteria.Alphaproteobacteria.Caulobacterales | 4.163 |
| Bacteria.Proteobacteria.Alphaproteobacteria.Caulobacterales.Caulobacteraceae | 3.559 |
| Bacteria.Proteobacteria.Alphaproteobacteria.Caulobacterales.Caulobacteraceae.Brevundimonas | 3.559 |
| Bacteria.Proteobacteria.Alphaproteobacteria.Caulobacterales.Caulobacteraceae.Brevundimonas.Otu000216 | 3.559 |
| Bacteria.Proteobacteria.Alphaproteobacteria.Rhizobiales | 3.412 |
| Bacteria.Proteobacteria.Alphaproteobacteria.Rhizobiales.Phyllobacteriaceae | 3.412 |
| Bacteria.Proteobacteria.Alphaproteobacteria.Rhizobiales.Phyllobacteriaceae.uc | 3.412 |
| Bacteria.Proteobacteria.Alphaproteobacteria.Rhizobiales.Phyllobacteriaceae.uc.Otu000057 | 3.483 |
| Bacteria.Proteobacteria.Alphaproteobacteria.Rhodobacterales | 4.889 |
| Bacteria.Proteobacteria.Alphaproteobacteria.Rhodobacterales.Rhodobacteraceae | 4.889 |
| Bacteria.Proteobacteria.Alphaproteobacteria.Rhodobacterales.Rhodobacteraceae.uc | 4.889 |
| Bacteria.Proteobacteria.Alphaproteobacteria.Rhodobacterales.Rhodobacteraceae.uc.Otu000006 | 4.742 |
| Bacteria.Proteobacteria.Alphaproteobacteria.Rhodobacterales.Rhodobacteraceae.uc.Otu000021 | 4.224 |
| Bacteria.Proteobacteria.Alphaproteobacteria.Rhodobacterales.Rhodobacteraceae.uc.Otu000045 | 3.635 |
| Bacteria.Proteobacteria.Alphaproteobacteria.Rhodobacterales.Rhodobacteraceae.uc.Otu000148 | 3.377 |
| Bacteria.Proteobacteria.Alphaproteobacteria.Sphingomonadales | 4.387 |
| Bacteria.Proteobacteria.Alphaproteobacteria.Sphingomonadales.Sphingomonadaceae | 4.324 |
| Bacteria.Proteobacteria.Alphaproteobacteria.Sphingomonadales.Sphingomonadaceae.Sphingopyxis | 4.213 |
| Bacteria.Proteobacteria.Alphaproteobacteria.Sphingomonadales.Sphingomonadaceae.Sphingopyxis.Otu000064 | 4.213 |
| Bacteria.Proteobacteria.Alphaproteobacteria.Sphingomonadales.Sphingomonadaceae.uc | 3.694 |
| Bacteria.Proteobacteria.Alphaproteobacteria.Sphingomonadales.Sphingomonadaceae.uc.Otu000165 | 3.694 |
| Bacteria.Proteobacteria.Alphaproteobacteria.Sphingomonadales.uc | 3.587 |
| Bacteria.Proteobacteria.Alphaproteobacteria.Sphingomonadales.uc.uc | 3.585 |
| Bacteria.Proteobacteria.Alphaproteobacteria.Sphingomonadales.uc.uc.Otu000173 | 3.586 |
| Bacteria.Proteobacteria.Betaproteobacteria.Burkholderiales.Alcaligenaceae | 3.525 |
| Bacteria.Proteobacteria.Betaproteobacteria.Burkholderiales.Alcaligenaceae.uc | 3.524 |
| Bacteria.Proteobacteria.Betaproteobacteria.Burkholderiales.Alcaligenaceae.uc.Otu000172 | 3.526 |
| Bacteria.Proteobacteria.Betaproteobacteria.Burkholderiales.Comamonadaceae.Hydrogenophaga | 3.926 |
| Bacteria.Proteobacteria.Betaproteobacteria.Burkholderiales.Comamonadaceae.Hydrogenophaga.Otu000009 | 3.956 |
| Bacteria.Proteobacteria.Betaproteobacteria.Burkholderiales.Comamonadaceae.uc.Otu000095 | 4.032 |
| Bacteria.Proteobacteria.Gammaproteobacteria.Xanthomonadales | 4.171 |
| Bacteria.Proteobacteria.Gammaproteobacteria.Xanthomonadales.Xanthomonadaceae | 4.171 |

**Additional file 6**: Linear discriminant analyses (using LEfSe) identified differentially abundant bacterial OTUs, comparing PE- and PS-associated assemblages. Analyses are based on OTUs (16S rRNA gene data) with an average relative abundance of ≥0.1%. Listed are significantly differentially abundant features from the individual datasets ‘Baltic’, ‘Warnow’ and ‘WWTP’, the polymer, which the corresponding taxon colonized differentially abundantly, and the logarithmized LDA values. uc:unclassified.

| **Differentially abundant feature** | **Polymer** | **log LDA** |
| --- | --- | --- |
| **Dataset Baltic** |  |  |
| Bacteria.Bacteroidetes.Flavobacteriia | PE | 4.699 |
| Bacteria.Bacteroidetes.Flavobacteriia.Flavobacteriales | PE | 4.699 |
| Bacteria.Bacteroidetes.Flavobacteriia.Flavobacteriales.Flavobacteriaceae | PE | 4.679 |
| Bacteria.Bacteroidetes.Flavobacteriia.Flavobacteriales.Flavobacteriaceae.Tenacibaculum | PE | 3.446 |
| Bacteria.Planctomycetes.OM190.uc.uc.uc.Otu000028 | PE | 3.857 |
| Bacteria.Proteobacteria.Alphaproteobacteria.Sphingomonadales | PE | 3.857 |
| Bacteria.Proteobacteria.Alphaproteobacteria.Sphingomonadales.Erythrobacteraceae | PE | 3.857 |
| Bacteria.Proteobacteria.Alphaproteobacteria.Sphingomonadales.Erythrobacteraceae.uc | PE | 3.587 |
| Bacteria.Proteobacteria.Alphaproteobacteria.Sphingomonadales.Erythrobacteraceae.uc.Otu000089 | PE | 3.587 |
| Bacteria.Bacteroidetes.Bacteroidia | PS | 4.176 |
| Bacteria.Bacteroidetes.Flavobacteriia.Flavobacteriales.Flavobacteriaceae.Aquibacter.Otu000044 | PS | 3.978 |
| Bacteria.Bacteroidetes.Flavobacteriia.Flavobacteriales.Flavobacteriaceae.uc.Otu000048 | PS | 3.730 |
| Bacteria.Bacteroidetes.Sphingobacteriia.Sphingobacteriales.Saprospiraceae.uc.Otu000037 | PS | 3.413 |
| Bacteria.Bacteroidetes.uc.uc.uc.uc.Otu000065 | PS | 3.694 |
| Bacteria.Planctomycetes | PS | 4.444 |
| Bacteria.Planctomycetes.Planctomycetacia | PS | 4.511 |
| Bacteria.Planctomycetes.Planctomycetacia.Planctomycetales | PS | 4.511 |
| Bacteria.Planctomycetes.Planctomycetacia.Planctomycetales.Planctomycetaceae | PS | 4.511 |
| Bacteria.Planctomycetes.Planctomycetacia.Planctomycetales.Planctomycetaceae.Planctomyces | PS | 4.409 |
| Bacteria.Planctomycetes.Planctomycetacia.Planctomycetales.Planctomycetaceae.Planctomyces.Otu000002 | PS | 4.265 |
| Bacteria.Planctomycetes.Planctomycetacia.Planctomycetales.Planctomycetaceae.Planctomyces.Otu000076 | PS | 3.596 |
| Bacteria.Planctomycetes.Planctomycetacia.Planctomycetales.Planctomycetaceae.Planctomyces.Otu000098 | PS | 3.641 |
| Bacteria.Planctomycetes.Planctomycetacia.Planctomycetales.Planctomycetaceae.uc | PS | 3.670 |
| Bacteria.Planctomycetes.Planctomycetacia.Planctomycetales.Planctomycetaceae.uc.Otu000096 | PS | 3.662 |
| Bacteria.Proteobacteria.Betaproteobacteria.Burkholderiales.Comamonadaceae.uc.Otu000142 | PS | 4.426 |
| Bacteria.Verrucomicrobia | PS | 3.809 |
| Bacteria.Verrucomicrobia.Verrucomicrobiae | PS | 3.831 |
| Bacteria.Verrucomicrobia.Verrucomicrobiae.Verrucomicrobiales | PS | 3.831 |
| Bacteria.Verrucomicrobia.Verrucomicrobiae.Verrucomicrobiales.Verrucomicrobiaceae | PS | 3.699 |
| Bacteria.Verrucomicrobia.Verrucomicrobiae.Verrucomicrobiales.Verrucomicrobiaceae.Haloferula | PS | 3.369 |
| Bacteria.Verrucomicrobia.Verrucomicrobiae.Verrucomicrobiales.Verrucomicrobiaceae.Haloferula.Otu000074 | PS | 3.495 |
| Bacteria.Verrucomicrobia.Verrucomicrobiae.Verrucomicrobiales.Verrucomicrobiaceae.Luteolibacter | PS | 3.602 |
| Bacteria.Verrucomicrobia.Verrucomicrobiae.Verrucomicrobiales.Verrucomicrobiaceae.Luteolibacter.Otu000116 | PS | 3.602 |
| Bacteria.Verrucomicrobia.Verrucomicrobiae.Verrucomicrobiales.Verrucomicrobiaceae.Prosthecobacter | PS | 3.247 |
| Bacteria.Verrucomicrobia.Verrucomicrobiae.Verrucomicrobiales.Verrucomicrobiaceae.Prosthecobacter.Otu000152 | PS | 3.247 |
| Bacteria.Verrucomicrobia.Verrucomicrobiae.Verrucomicrobiales.Verrucomicrobiaceae.uc | PS | 3.268 |
| Bacteria.Verrucomicrobia.Verrucomicrobiae.Verrucomicrobiales.Verrucomicrobiaceae.uc.Otu000103 | PS | 3.268 |
| **Dataset Warnow** |  |  |
| Bacteria.Planctomycetes.Planctomycetacia.Planctomycetales.Planctomycetaceae.Pirellula | PE | 3.696 |
| Bacteria.Planctomycetes.Planctomycetacia.Planctomycetales.Planctomycetaceae.Pirellula.Otu000091 | PE | 3.701 |
| Bacteria.Bacteroidetes.Flavobacteriia.Flavobacteriales.uc | PS | 3.371 |
| Bacteria.Bacteroidetes.Flavobacteriia.Flavobacteriales.uc.uc | PS | 3.371 |
| Bacteria.Bacteroidetes.Flavobacteriia.Flavobacteriales.uc.uc.Otu000040 | PS | 3.153 |
| Bacteria.Proteobacteria.Gammaproteobacteria.Cellvibrionales.Cellvibrionaceae.uc.Otu000018 | PS | 3.281 |
| Bacteria.Verrucomicrobia.Verrucomicrobiae.Verrucomicrobiales.Verrucomicrobiaceae.Roseibacillus | PS | 2.831 |
| Bacteria.Verrucomicrobia.Verrucomicrobiae.Verrucomicrobiales.Verrucomicrobiaceae.Roseibacillus.Otu000097 | PS | 2.850 |
| **Dataset WWTP** |  |  |
| Bacteria.Proteobacteria.Betaproteobacteria.Burkholderiales.Comamonadaceae.Hydrogenophaga.Otu000009 | PE | 4.096 |

**Additional file 7**: Enumeration of *Vibrio* sp. in wood-, PE-, PS-associated assemblages, particle-attached (PAW) and free-living (FLW) water communities, as determined via *Vibrio*-targeting qPCR. Given are sample source and station as well as replicate number and gene copies per ng.

| **Source** | **Station** | **Replicate** | **Gene copies / ng** |
| --- | --- | --- | --- |
| Wood | 2 | 3 | 2.16E+08 |
| Wood | 2 | 2 | 2.06E+07 |
| Wood | 2 | 1 | 1.30E+07 |
| FLW | 1 | 2 | 3.27E+06 |
| PAW | 1 | 2 | 1.69E+06 |
| Wood | 5 | 2 | 7.97E+05 |
| Wood | 5 | 1 | 5.88E+05 |
| Wood | 4 | 1 | 3.28E+05 |
| FLW | 7 | 1 | 2.70E+05 |
| PAW | 5 | 2 | 2.67E+05 |
| PAW | 4 | 1 | 2.27E+05 |
| PAW | 4 | 2 | 2.07E+05 |
| Wood | 4 | 2 | 1.59E+05 |
| FLW | 4 | 1 | 1.16E+05 |
| PAW | 7 | 1 | 1.03E+05 |
| FLW | 7 | 2 | 8.48E+04 |
| PS | 4 | 1 | 6.95E+04 |
| PAW | 7 | 2 | 6.62E+04 |
| PAW | 2 | 2 | 5.16E+04 |
| PE | 5 | 1 | 3.58E+04 |
| PAW | 5 | 1 | 3.45E+04 |
| FLW | 1 | 1 | 1.96E+04 |
| PS | 2 | 1 | 1.46E+04 |
| PAW | 1 | 1 | 1.14E+04 |
| FLW | 2 | 1 | 9.84E+03 |
| PAW | 2 | 1 | 8.27E+03 |
| PE | 1 | 1 | 4.54E+03 |
